# Acceptability and feasibility of research grade wearables for monitoring heat stress in Kenyan farmers

**DOI:** 10.1038/s41746-025-01601-6

**Published:** 2025-05-07

**Authors:** Daniel Kwaro, Stefan Mendt, Julius Okoth, Stephen Munga, Hanns-Christian Gunga, Zoë Hannah Heim, Ina Matzke, Aditi Bunker, Sandra Barteit, Martina Anna Maggioni

**Affiliations:** 1 Charité—Universitätsmedizin Berlin, Institute of Physiology, Center for Space Medicine and Extreme Environment Berlin, Berlin, Germany; 2 Kenya Medical Research Institute, Kisumu, Kenya; 3 Heidelberg Institute of Global Health (HIGH), Faculty of Medicine and University Hospital, Heidelberg University, Heidelberg, Germany; 4 Department of Biomedical Sciences for Health, Università degli Studi di Milano, Milano, Italy

**Keywords:** Climate-change impacts, Occupational health, Developing world

## Abstract

Sub-Saharan Africa faces increasing heat events due to climate change, affecting health and productivity. Wearable technology, though promising for monitoring these impacts, is underexplored in this region. This pilot study evaluated the acceptability and feasibility of research-grade wearables for monitoring heat stress among Kenyan subsistence farmers. In Siaya, 48 farmers (50% women) were monitored for 14 days using sensors to measure heart rate, core temperature, sleep, activity, and geo-location, alongside environmental data loggers for wet bulb globe temperature. Participants mostly rated their experience on a 5-point Likert scale and provided additional non-Likert feedback, with over 95% reporting high device likability and minimal disruption. Data availability was 88% for actigraphy and 100% for core temperature, with a median completeness of 100% for most devices. Women experienced greater heat strain than men. These findings demonstrate that research-grade wearables are acceptable and feasible for real-time heat stress monitoring in rural Africa.

## Introduction

A growing body of evidence indicates that climate change exacerbates the frequency and intensity of high temperatures in sub-Saharan Africa (SSA), affecting outdoor workers such as farmers^1,2^. The combination of extreme heat and physical labor raises an individual’s heat load, which refers to the total heat gained from external sources (e.g., temperature and solar radiation) and internal sources (i.e., metabolic heat)^3^. This increased heat load can lead to heat stress, a condition in which there is increased demand on the body’s thermoregulatory system to dissipate excess heat and maintain core body temperature (CBT) at 37 ± 5 °C^4^. Prolonged heat stress reduces residual work capacity—the remaining ability to perform tasks before physiological limits—while also increasing the risk of heat-related illnesses such as heat exhaustion and heat stroke, and compromises occupational safety^5^. Additionally, heat stress disrupts sleep, impairing recovery and further diminishing work capacity. Farmers in SSA are already vulnerable due to limited financial resources and access to adaptation technologies such as mechanization and cooling solutions; therefore, they face an increasing risk of worsening poverty and economic insecurity^2^.

To effectively address the impact of climate-induced heat stress, it is essential to monitor both environmental conditions and individual physiological responses^5^. Wearable technologies enable real-time personal monitoring of cardiovascular strain, such as increased heart rate (HR) and thermal strain, as indicated by rising core body temperature (CBT)^6^. These responses to heat stress, collectively known as heat strain, represent the body’s attempt to restore heat balance. By integrating HR and CBT data, wearables can provide a comprehensive measure of overall physiological strain through indices such as the Physiological Strain Index (PSI)^7^. Additionally, wearables track the intensity of physical activity and sleep quality, offering valuable insights into physical performance and recovery^8^. In addition to health risk monitoring, wearable devices are versatile tools that have been used across a variety of healthcare domains to facilitate diagnosis, track disease progression, and assess patient responses to interventions^8,9^. On the other hand, environmental data loggers can monitor factors that affect heat dissipation, such as air temperature, humidity, wind speed, and solar radiation^4^. These indicators are often combined into indices such as the Wet-Bulb Globe Temperature (WBGT), which is widely used to assess environmental heat stress^10^. Integrating individual and environmental data offers a powerful opportunity to understand heat stress more comprehensively, yet such approaches are underutilized in SSA; moreover, research on wearables in the region is scarce^6,11^. An investigation of the acceptability, feasibility, and practical application of these methods for data collection is particularly lacking. In addition, existing studies primarily involve consumer-grade wearables, with few studies making use of research-grade wearables.

Personal monitoring with consumer- or research-grade wearables involves continuous or periodic tracking of physiological and environmental parameters in real time^8^. Research-grade wearables, such as the Tcore CBT sensor, are designed for research and have been validated for accuracy under extreme conditions^12^. Furthermore, while consumer-grade wearables may use proprietary algorithms to provide simplified data outputs, research-grade wearables retain raw sensor data, allowing researchers to apply custom algorithms for specific analyses^13^. In addition, consumer-grade wearables, such as Fitbit and Apple Watch, are geared toward fitness and lifestyle tracking, offering less accuracy but widespread use due to affordability and ease of use^13–15^. A study by Huhn et al. found consumer-grade wearables to be feasible and acceptable among rural African farmers, consistent with global findings^16^. While these devices are widely used in Western countries and increasingly in low-resource settings^11^, research-grade wearables have not yet undergone broad adoption. Bridging the evidence gap on the acceptability and feasibility of both research-grade wearables and environmental sensors is essential for facilitating their wider adoption in SSA.

In SSA, Health and Demographic Surveillance Systems (HDSSs), which include large population-based cohorts, offer a platform for incorporating research-grade wearables into climate research data. One such example is the KEMRI/CDC HDSS in western Kenya, which regularly collects morbidity data from a cohort with a current mid-year population of approximately 250,000^17^. Integrating this morbidity information with data from wearables and environmental sensors enables detailed heat stress analysis. However, challenges such as infrastructure limitations and lack of wearables persist in most HDSSs^18^. In response, a recent research unit (FOR 2936: ‘Climate Change and Health in Sub-Saharan Africa’), funded by the German Research Foundation (DFG), has been addressing these issues since 2020, among others^19^. The research unit has facilitated the installation of automated weather stations and the deployment of various consumer- and research-grade wearables at the KEMRI/CDC HDSS. The feasibility study presented in this paper is a component of the “Climate change, heat stress, and their impact on health and working capacity” subproject, which evaluates the impact of weather extremes on the health and residual working capacity of subsistence farmers at the KEMRI/CDC HDSS. Using research-grade wearables and portable sensors, several parameters, including physiological parameters (such as heart rate and core body temperature), physical activity, sleep quality, geographic location, and indoor conditions, were monitored. The specific inclusion of such physiological parameters, along with indoor/outdoor environmental factors (i.e., the WBGT index), is specifically designed to quantify heat stress and related reductions in labor performance and capacity. This study is one of the first to simultaneously collect physiological, behavioral, and environmental data using research-grade wearables and data loggers in a real-life setting in SSA.

As mentioned above, the use of research-grade sensors deployed as wearables and environmental data loggers in SSA remains underexplored. This study seeks to fill that gap by evaluating the feasibility and acceptability of these technologies in a rural Kenyan setting. Assessing feasibility is essential to ensure these devices are functional, culturally appropriate, and practical in contexts where socio-cultural and environmental factors may influence usability. Without such assessments, there is a risk of low adoption and wasted resources. By identifying adoption challenges, feasibility studies help ensure that wearables provide high-resolution data to quantify heat stress impacts, and inform targeted interventions for vulnerable populations. These findings will lay the groundwork for their broader use in future studies, helping to examine the effects of environmental conditions on individual work capacity and health, assess population-level risks, and identify necessary interventions^5^.

## Results

A total of 48 participants were recruited for the study, with 20 participants allocated to Group 1 and 28 participants allocated to Group 2. There was complete follow-up of all participants, without dropouts. Table 1 provides an overview of the participants’ anthropometric characteristics, revealing sex-related differences in age, weight, height, and fat mass.

**Table 1:** Characteristics of study participants

	Overall	Women	Men	*p*-value
	*n* = 48 mean (SD)	*n* = 24 mean (SD)	*n* = 24 mean (SD)	
Age (years)	28.6 (4.7)	26.1 (3.6)	31.1 (4.42)	<0.001
Height (cm)	166.4 (8.5)	161.2 (7.1)	171.4 (6.55)	<0.001
Weight (kg)	59.8 (9.7)	55.1 (9.1)	64.3 (8.19)	0.001
BMI (kg/m^2^)	21.5 (2.9)	21.1 (2.6)	21.9 (3.16)	0.356
TBW (L)	33.2 (5.8)	28.2 (3.46)	37.8 (3.33)	<0.001
FM (%)	14.8 (5.2)	17.0 (4.87)	12.7 (4.75)	0.004

Anthropometric and body composition data from the study sample.

*BMI* body mass index, *TBW* - percentage of total body water, *FM* fat mass.

### Acceptability of wearables

**Fig. 1:**
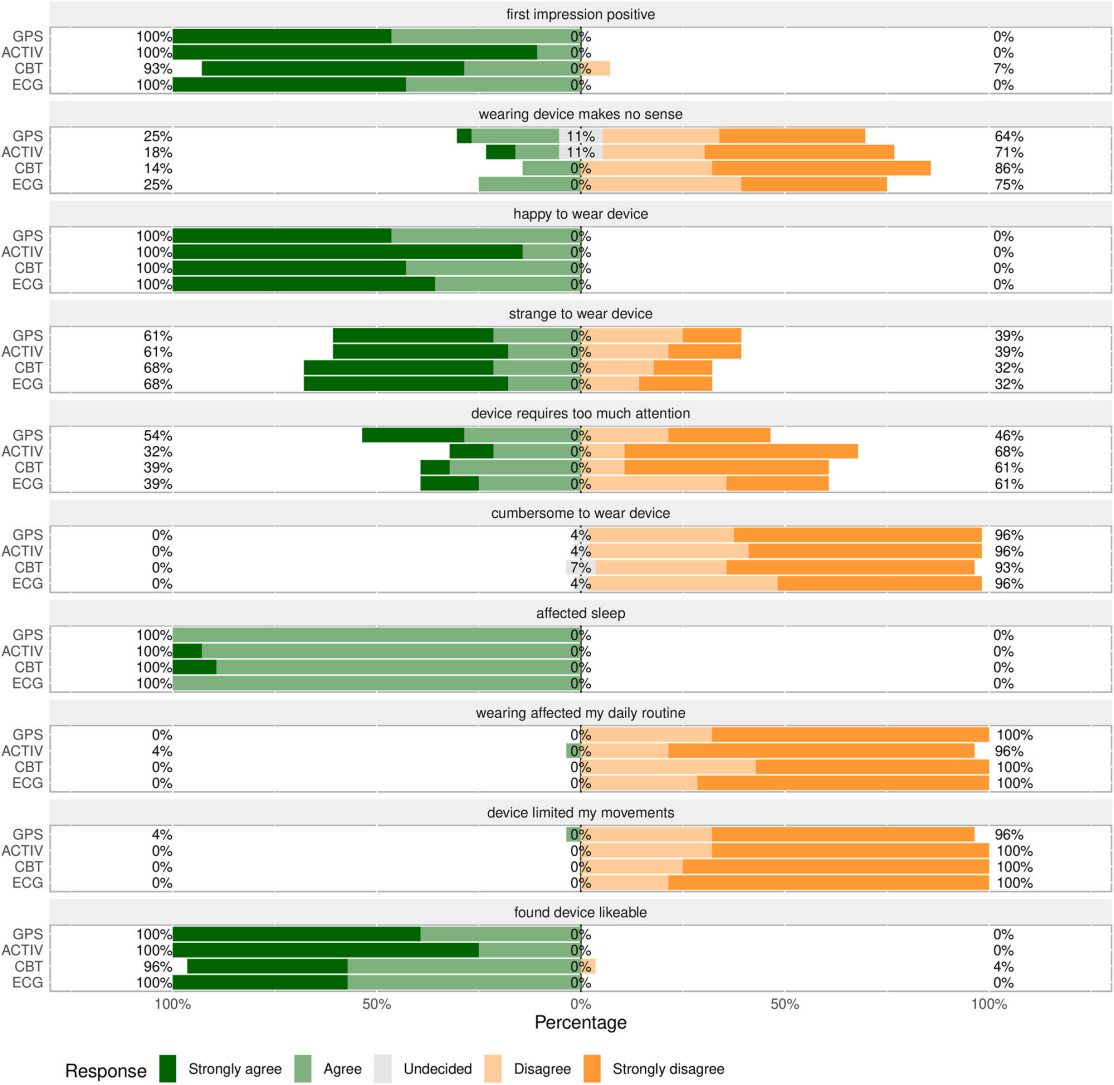
Acceptability ratings for wearables. This divergent bar graph illustrates overall sentiment ratings for representative Likert scale items. The percentage labels indicate the distribution of responses across the high (strongly agree, agree), neutral (undecided), and low (strongly disagree, disagree) segments of the scale. The wearables are identified by the parameters they recorded: CBT (core body temperature), ECG (electrocardiography), ACT (actigraphy), and GPS (location tracking with a Global Positioning System device).

Figure 1 illustrates the acceptability ratings for the four wearable devices: the electrocardiography (ECG), core body temperature (CBT), actigraphy (ACT), and global positioning system (GPS) devices. Before use, participants showed a high level of agreement on a positive first impression of ECG, ACT, and GPS at 100%, with CBT closely followed at 93%. In terms of the perceived usefulness of the wearable devices, only a minority of the participants agreed that wearing them made no sense: 25% for both ECG and GPS, 18% for ACT, and 14% for CBT. Regarding the individual’s psychological response, all participants expressed happiness while wearing them. However, a significant proportion (68%) of participants found ECG and CBT wearables strange to wear. Regarding the cognitive burden of using a wearable device, varying percentages of participants agreed that their devices required too much attention: 61% for both ECG and CBT, 68% for ACT, and 46% for GPS. When assessing the physical burden related to the wearables, a minority of participants found the devices cumbersome to wear, with only 4% or fewer reporting such issues. Additionally, 4% of participants agreed that the wearable GPS devices limited their movements, while none reported such constraints for the other wearable devices. For the impact on daily routines, only 4% of participants agreed that the ACT wearable device affected their daily routines, while none reported such effects for the other wearable devices. Furthermore, all participants agreed that wearing wearable devices had some impact on their sleep. Lastly, in terms of aesthetic appeal, all participants found the ECG, GPS, and CBT wearable devices likable, with 96% sharing this sentiment for ACT.

**Fig. 2:**
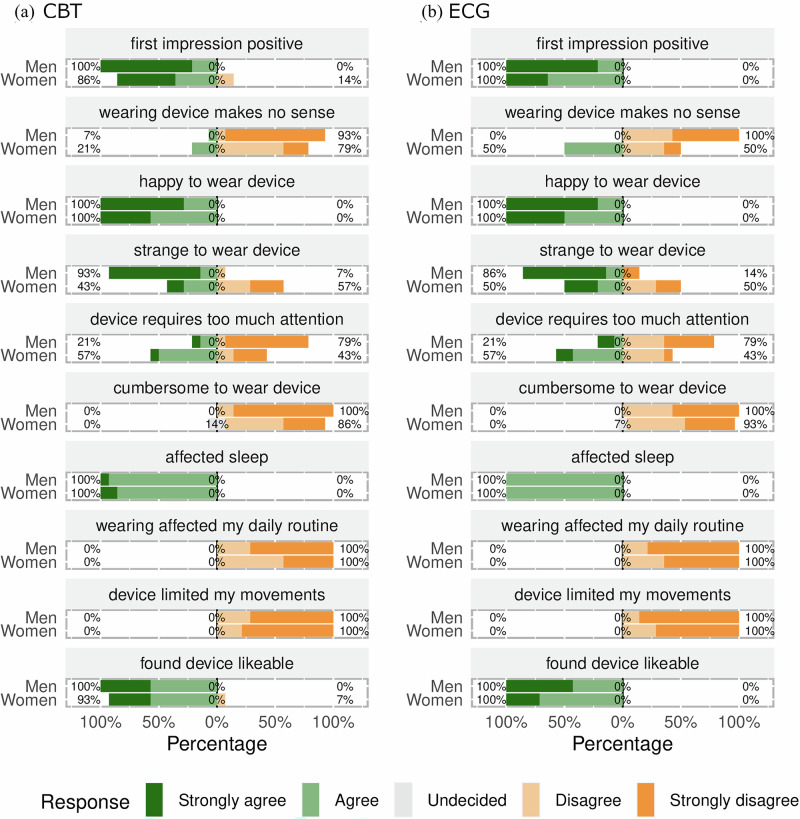
Sex-stratified acceptability ratings for CBT and ECG wearables. These divergent bar graphs illustrate sentiment ratings, stratified by sex, for representative Likert scale items. The percentage labels indicate the distribution of responses across the high (strongly agree, agree), neutral (undecided), and low (strongly disagree, disagree) segments of the scale. Panel (**a**) illustrates experiences with the wearable core body temperature (CBT) measurement device, while panel (**b**) focuses on the wearable electrocardiography (ECG) device.

**Fig. 3:**
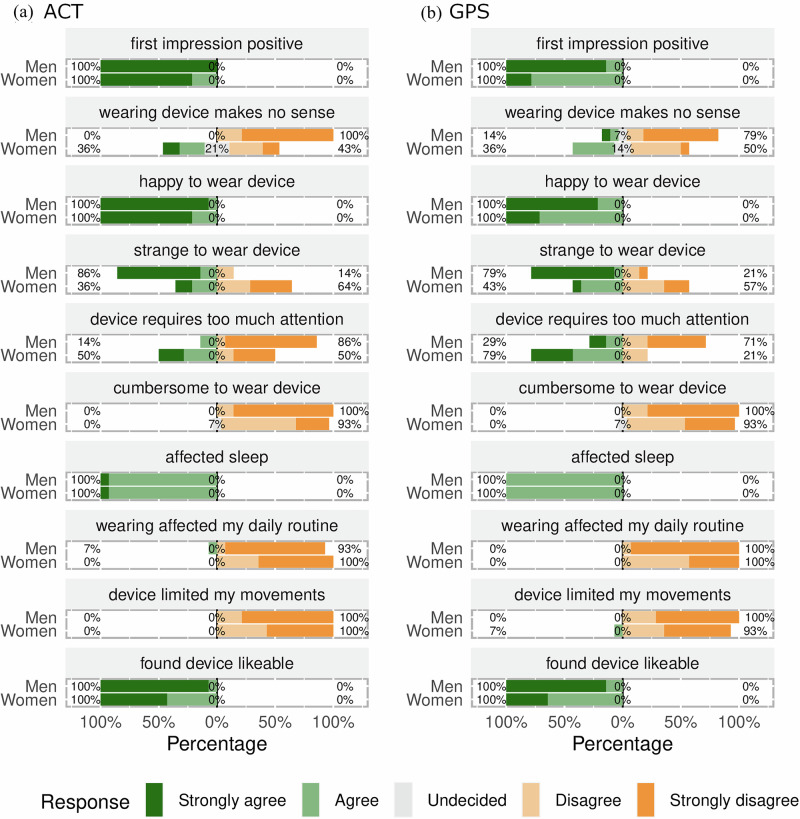
Sex-stratified acceptability ratings for ACT and GPS wearables. These divergent bar graphs illustrate sentiment ratings, stratified by sex, for representative Likert scale items. The percentage labels indicate the distribution of responses across the high (strongly agree, agree), neutral (undecided), and low (strongly disagree, disagree) segments of the scale. Panel (**a**) illustrates experiences with the actigraphy (ACT) wearable device, while panel (**b**) focuses on the global position system (GPS) tracking wearable device.

There was a notable variation in acceptability between sexes, as depicted in Figs. 2 and 3. A higher percentage of women expressed that the purpose of the wearables made no sense, ranging from 21% for CBT to 50% for ECG. On the contrary, the percentage of men who reported that wearables made no sense was generally very low, ranging from 0% to 14%. Men were more likely than women to perceive wearing the wearables as strange, with this difference particularly pronounced for the CBT device (93% for men vs. 43% for women). Additionally, a higher proportion of women reported that wearables required too much attention, especially for GPS (79% for women vs. 29% for men). However, other aspects of acceptability were similar between the sexes, with minimal or no differences in positive vs. negative sentiments.

Table 2 summarizes the social interactions experienced by the study participants while wearing various wearable devices. Overall, CBT wearables attracted the most public interest, with 29% of individuals being asked about them and 54% of conversations involving them. On the contrary, the ACT wearable had the lowest percentage of inquiries (20%), and the GPS wearable was the least discussed (32%). Compared to men, a higher proportion of women reported public inquiries and discussions about each wearable device. It is important to note that the social interaction questions were administered to only 14 pairs of men and women. An erroneous skip pattern in the CAPI excluded the questions in the first run of the study.

**Table 2:** Social experiences with wearable devices

	Social experience	Women (*N* = 14)	Men (*N* = 14)	Overall (*N* = 28)
ECG	People asked about device	4 (29%)	2 (14%)	6 (21%)
	Device was topic of conversation	9 (64%)	2 (14%)	11 (39%)
CBT	People asked about device	5 (36%)	3 (21%)	8 (29%)
	Device was topic of conversation	9 (64%)	6 (43%)	15 (54%)
ACT	People asked about device	8 (29%)	3 (11%)	11 (20%)
	Device was topic of conversation	15 (54%)	11 (39%)	26 (46%)
GPS	People asked about device	6 (43%)	1 (7%)	7 (25%)
	Device was topic of conversation	7 (50%)	2 (14%)	9 (32%)

The descriptive table presents the number of individuals, both overall and by sex, reporting public inquiries and discussions about each device, with quantities and in brackets respective percentages for each group (women, men, and overall). Acronyms for wearables: *ECG* electrocardiography, *CBT* core body temperature measurement, *ACT* actigraphy, *GPS* global positioning system.

Note: The total number of participants was 28, as these questions were asked during the second run of the study only (including 14 pairs of men and women, instead of 24 pairs).

### Feasibility of individual and environmental monitoring

**Fig. 4:**
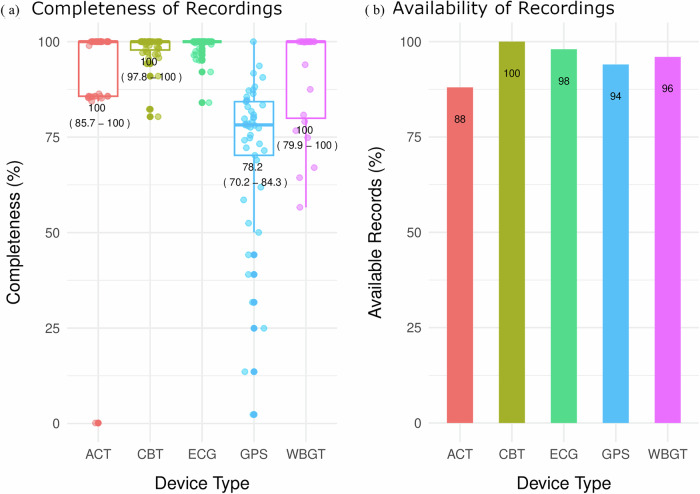
Summary statistics on the availability and completeness of good-quality data across devices. This multipanel plot comprises panel (**a**), which displays the distribution of individual-level percentages of good-quality data recordings across various devices in a boxplot format, annotated with medians and interquartile ranges (IQRs, Q1–Q3). Panel (**b**) presents a bar plot illustrating the percentage of individuals (for wearables) or households (for which WBGT data were used) with at least one data point for each type of device. The wearables and data logger used are identified by the parameters they recorded: ACT (actigraphy), CBT (core body temperature), ECG (electrocardiography), GPS (location tracking with a Global Positioning System device), and WBGT (wet-bulb globe temperature).

Figure 4 shows summary statistics of data availability and completion rates across different wearables. Notably, only 88% of the people had at least one good-quality ACT recording available, marking the lowest availability among the wearables. However, more than 94% of the remaining wearables had one or more good-quality recordings. The median data completeness rate was 100% for all wearables except the GPS wearable device, for which the median completeness rate was 79%. Considerable variations in the duration of recorded data deemed to be of good quality were observed, particularly for the wearable GPS devices, which had the widest interquartile range (median 79%, IQR 70%-84%). Although the recordings were incomplete in some cases, as described above, the study staff reported no challenges with operational procedures (e.g., device configuration, donning, data saving, and data synchronization). Furthermore, no wearables were lost or damaged throughout the study period.

**Fig. 5:**
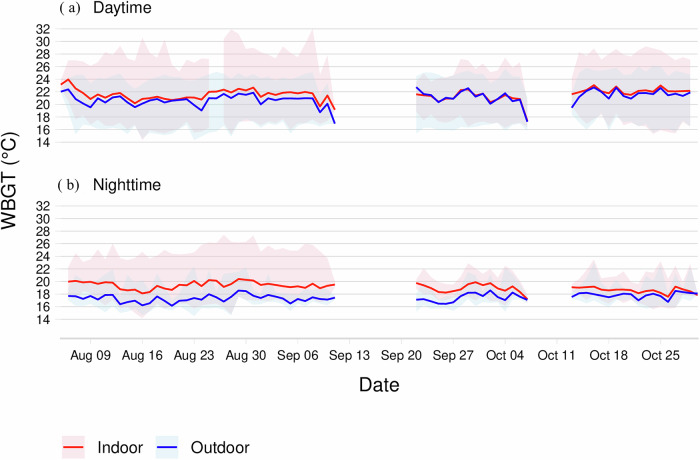
Daily WBGT variation by location and time of day. This multipanel plot shows the mean WBGT (lines) in degrees Celsius (°C) and its daily range (shaded areas) for outdoor (dark blue) and indoor (pink) settings, separated into daytime (**a**) and nighttime (**b**) panels. WBGT wet-bulb globe temperature. The daily (aggregating day and night) WBGT for the whole period of recording was significantly greater indoors than outdoors (20.5 °C vs. 19.3 °C, *p* < 0.05).

**Fig. 6:**
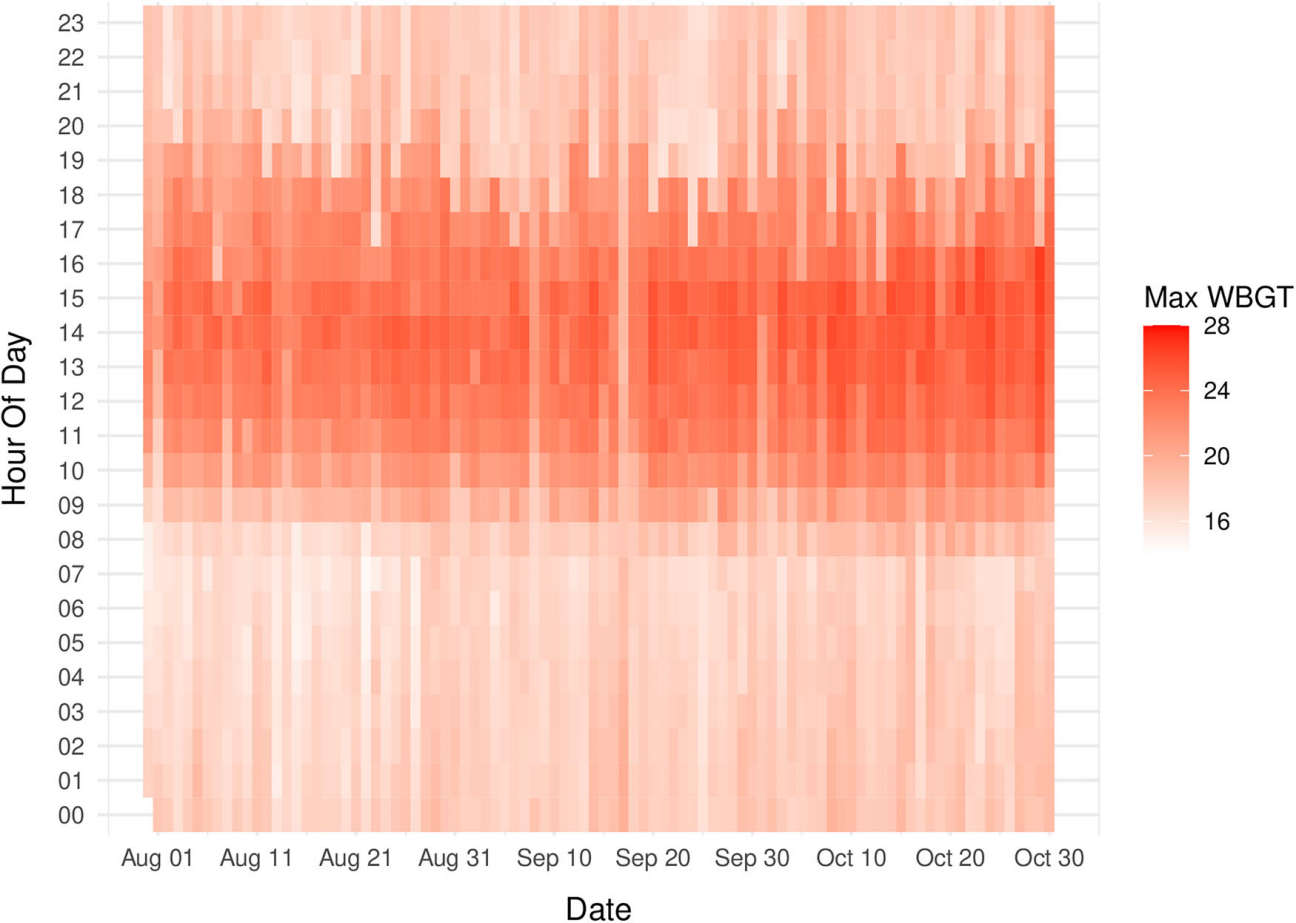
Daily outdoor WBGT maximum values. Heat map showing the maximum WBGT reached during each hour of the day throughout the study period. The color scale indicates WBGT intensity from lowest (lightest) to highest (darkest red). Each cell represents the maximum value recorded during that specific hour of the given day.

In terms of the external thermal environment, the daily mean WBGT, when aggregating day and night values, was significantly higher indoors than outdoors (20.5 °C vs. 19.3 °C, *p* < 0.05), as shown in Fig. 5. In Fig. 6, the daily hourly variation in the maximum outdoor WBGT over the study period is shown, increasing from approximately 8:00, peaking between 14:00 and 15:00, and then decreasing around 19:00  at dusk.

Table 3 presents the physiological indicators of heat-related strain (mean ± SD) and compares the overall Physiological Strain Index (PSI), cardiac and thermal components of heat-related strain, and physical activity and sleep patterns between men and women. Women had significantly higher PSI than men did (3.28 ± 0.72 vs. 2.9 ± 0.88, *p* = 0.010). Cardiac indices revealed that women had a higher baseline heart rate (51.66 ± 4.94 vs. 47.94 ± 7.35 bpm, *p* = 0.050), a greater relative increase in heart rate (34.86 ± 13.09% vs. 30.95 ± 12.9%, *p* = 0.010), and a higher peak heart rate (99.28 ± 18.35 vs. 90.75 ± 19.54 bpm, *p* < 0.001), whereas men had a greater heart rate reserve capacity (138.76 ± 8.32 vs. 136.63 ± 5.58 bpm, *p* < 0.001). Thermal indices revealed that women had a slightly higher resting core body temperature (36.11 ± 0.14 °C vs. 36.07 ± 0.11 °C, *p* = 0.039), but there were no significant differences in mean core body temperature (37.1 ± 0.41 °C vs. 37.0 ± 0.48 °C, *p* = 0.353) or relative change in CBT (29.48 ± 10.63% vs. 27.28 ± 13.31%, *p* = 0.395).

**Table 3:** Comparisons of physiological activity, physical activity, and sleep indices between men and women

Parameter	Women (*n* = 24) Mean ± SD	Men (*n* = 24) Mean ± SD	*p*-value
**Cardiovascular indicators**			
Heart rate (bpm)	82.52 ± 14.91	74.97 ± 16.79	0.010
Coefficient of variation in HR (%)	5.03 ± 1.9	4.87 ± 2.05	0.610
Resting heart rate (bpm)	51.66 ± 4.94	47.94 ± 7.35	0.050
Peak heart rate (bpm)	99.28 ± 18.35	90.75 ± 19.54	0.000
Heart rate reserve capacity (bpm)	136.63 ± 5.58	138.76 ± 8.32	0.000
Relative change in HR (%)	34.86 ± 13.09	30.95 ± 12.9	0.010
**Thermal indicators**			
CBT (°C)	37.1 ± 0.41	37 ± 0.48	0.353
Resting CBT (°C)	36.11 ± 0.14	36.07 ± 0.11	0.039
Peak CBT (°C)	37.97 ± 0.42	38.13 ± 0.47	0.569
CBT reserve capacity (°C)	3.39 ± 0.14	3.43 ± 0.11	0.000
Relative change in CBT (%)	29.48 ± 10.63	27.28 ± 13.31	0.395
**Overall physiological strain indicator**			
Physiological Strain Index (PSI)	3.28 ± 0.72	2.9 ± 0.88	0.010
**Physical activity indicators**			
Daily MVPA (hours)	3.0 ± 2.0	2.3 ± 1.9	0.408
Daily steps (count)	11, 290 ± 6, 216	10, 125 ± 8,454	0.863
Daily distance (km)	11.7 ± 6.3	9.3 ± 4.5	0.398
**Sleep pattern indicators**			
Total bedtime (hours)	8.4 ± 5.3	9.1 ± 5.3	0.337
Total sleep time (hours)	4.6 ± 2.1	4.9 ± 2.5	0.560
Sleep efficiency (%)	61.8 ± 24.0	59.7 ± 26.7	0.895

The table presents the mean and standard deviation (SD) values for the Physiological Strain Index (PSI), cardiac strain indicators (including heart rate [HR], thermal strain indicators (including core body temperature [CBT], physical activity (including moderate-to-vigorous physical activity [MVPA] time) and sleep patterns*. p*-values were obtained using a linear mixed-effects model (LME) with the indicator as the response variable, sex as the independent variable, and individual as a random effect to account for within-subject variability.

**Fig. 7:**
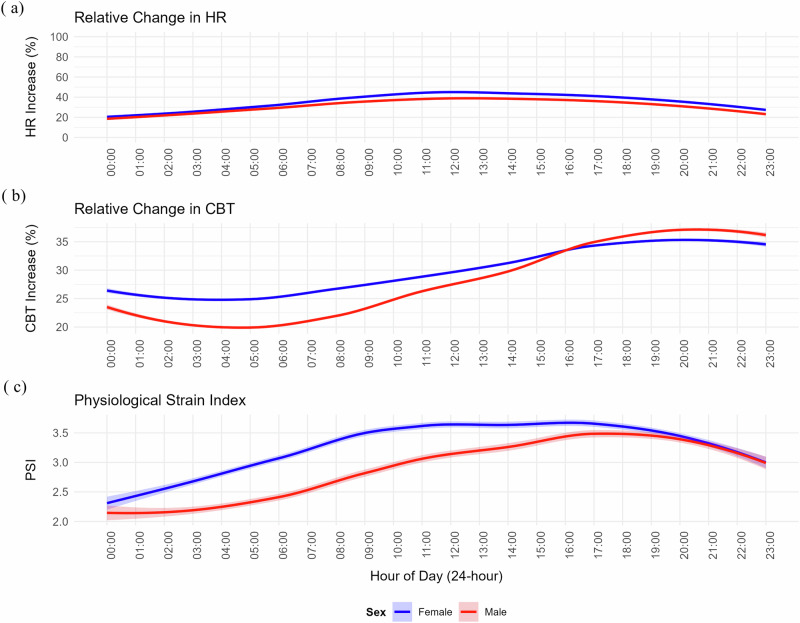
24-Hour Time Series of HR, CBT, and PSI by Sex. Panel (**a**) shows the relative change in heart rate (HR) over the course of the day, calculated as the percentage increase relative to the individual’s heart rate reserve (HRR). Panel (**b**) shows the relative change in core body temperature (CBT), calculated as the percentage increase relative to the individual’s CBT reserve capacity. Panel (**c**) displays the Physiological Strain Index (PSI), a composite measure of heat strain, which reflects the combined impact on heart rate and CBT. In all panels, females (blue) and males (red) are compared across different times of day.

Figure 7 visualizes the 24-hour time series of these physiological indicators by sex. The relative change in heart rate (Fig. 7a) rises steadily for both sexes, beginning at midnight and peaking in the late afternoon, with females consistently showing higher HR values that remain elevated for a longer period. In terms of core body temperature (Fig. 7b), the increase in CBT starts at approximately 4:00 a.m. and follows a similar pattern, with females exhibiting slightly higher values throughout the day, peaking later in the afternoon, while men peak in the evening. Finally, the physiological strain index (Fig. 7c) indicated that females experienced greater overall strain, with a sharper increase in the PSI and higher peak values than males. Both sexes experience peak physiological strain in the afternoon, but females endure higher amounts of strain for longer periods of time.

**Fig. 8:**
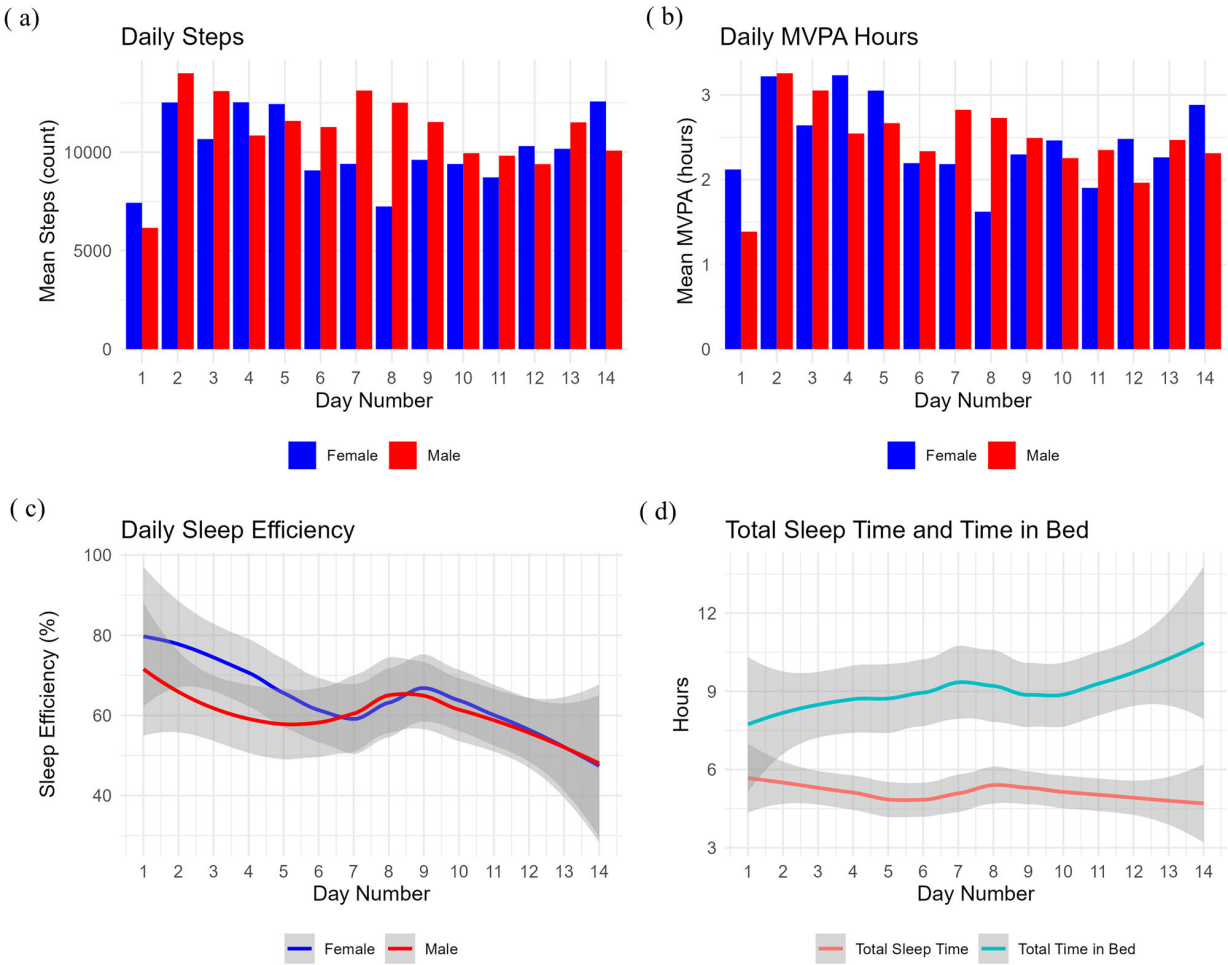
Daily trends in physical activity and sleep metrics over 14 days, stratified by sex. A multipanel plot showing daily trends in physical activity and sleep metrics over 14 days stratified by sex. Panel (**a**) displays a bar plot of the mean daily steps for males (red) and females (blue). Panel (**b**) shows a bar plot of the mean moderate-to-vigorous physical activity (MVPA) hours for males and females. Panel (**c**) presents a smoothed line plot of daily sleep efficiency (%) for males and females, with shaded regions representing confidence intervals. Panel (**d**) features a smoothed line plot comparing total sleep time (red) and total time in bed (teal). The 14-day periods did not necessarily overlap for all individuals, as participants were recruited on different days.

With respect to physical activity, women spent more time performing moderate-to-vigorous physical activity (MVPA) than men did (3.0 ± 2.0 vs. 2.3 ± 1.9 h, *p* = 0.408), although the difference was not statistically significant (Table 3). Women also had a higher average daily step count (11,290 ± 6,216 vs. 10,125 ± 8,454 steps, *p* = 0.863) and greater daily distance (11.7 ± 6.3 km vs. 9.3 ± 4.5 km, *p* = 0.398); however, neither difference reached statistical significance. In terms of sleep patterns, there were no significant differences between the sexes in total bedtime, total sleep time, or sleep efficiency. The 14-day trend for these indicators is illustrated in Fig. 8. The 14-day periods varied among participants since recruitment occurred on different days.

Supplementary Figures 1, 2, and 3, show data from a single female participant and illustrate the feasibility of collecting synchronized physiological, activity, and sleep data in response to environmental changes. Specifically, Supplementary Fig. 1 shows 24-hour records of the indoor and outdoor WBGT, CBT, and HR, with data averaging at 5-min intervals. On the other hand, Supplementary Fig. 2 shows 14-day actigraphy data, detailing her daily activity intensity, step count, total sleep time, and sleep efficiency, while Supplementary Fig. 3 visualizes her movements as tracked via GPS, highlighting a 3.3 km route spanning more than 5.5 hours, with 3.5 hours spent at the farms.

Supplementary Table 1 shows the fixed and random effect estimates from the mixed-effects model for predicting PSI. The outdoor WBGT was positively associated with the PSI (*β* = 0.32, *p* < 0.001), while sex (male) (*β* = 5.33, *p* < 0.001) and total MVPA hours (*β* = −0.16, *p* = 0.002) were also significant predictors. The interaction between outdoor WBGT and sex (male) (*β* = -0.27, *p* < 0.001) indicated that WBGT had a smaller effect on males.

## Discussion

In this study, we assessed the acceptability and feasibility of research-grade wearables in rural Kenya. Our study showed that research-grade wearables are well received, with more than 90% of participants perceiving them as useful, nondisruptive of their daily routine, physically comfortable, and aesthetically pleasing. Nevertheless, some minor discomforts regarding the attention drawn by the wearables in public and the amount of attention needed were reported. In addition, the study staff reported no difficulties with device procedures, and we observed high data availability and completion rates.

The positive initial impression reported by more than 90% of participants across all wearables (Figs. 1, 2, and 3) reflects strong acceptance and enthusiasm for these novel technologies. This indicates readiness to engage with technology and provides a promising foundation for successful implementation and adoption. Additionally, the majority of participants responded positively to questions about the key variables for user motivation that drive acceptance of new technologies as per the Technology Acceptance Model: high perceived benefits and zero or minimal burden during use^20^. A high perceived usefulness (benefit) implies a high degree of response efficacy, which is the individual belief that technology will help achieve a desired outcome. To this end, Din et al. (2020), in a paper focusing on frailty in elderly people in rural Tanzania, found that the acceptability of wearables was linked to expectations of personal benefits in terms of receiving a diagnosis or treatment^21^. However, given that our study enrolled healthy adult farmers, their motivation likely stemmed from collective benefit rather than individual gains, as they understood that this would reveal the burden of heat stress in their community and facilitate the development of adaptation strategies. The notion of collective benefit as motivation is particularly significant in the context of health research in rural settings, where community-oriented approaches are often more effective and well received^22^. Furthermore, the minimal burden of physical discomfort and disruption to daily routines, as reported by the majority of participants, indicates a high degree of self-efficacy, that is, the individual’s confidence in effectively using the technology. The high acceptability of research-grade wearables observed in this study aligns with similar findings on consumer-grade wearables in comparable contexts. For example, Huhn et al.’s study in Burkina Faso in 2022^16^ showed that consumer-grade wearables were highly acceptable and practical for producing useful longitudinal individual-level data in low-resource rural settings. The study revealed that more than 95% of the 140 participants had no issues with a wrist-worn actimetry device or a thermometry patch placed under the armpit, both of which were worn simultaneously.

While the study revealed positive findings, some challenges emerged, offering a multifaceted view of wearable technology acceptance in rural Kenya. These issues are more pronounced with CBT and ECG wearables. CBT devices, which are noticeable as headbands, have attracted significant public interest, especially among men (Table 2). However, heat-flux sensors on the forehead are currently the most suitable noninvasive way to monitor CBTs continuously. Even though questions on social reactions toward participants wearing wearables were administered to only 14 out of the 24 participant pairs in the study (see Table 3), we consider such results relevant, as they offer valuable insights into how technology use may increase social burden. This understanding is crucial for comprehending wearable technology acceptance in community settings where adherence to social norms is essential. With regard to the ECG wearables, women were particularly concerned that they required too much attention when worn. They may have felt the need to constantly monitor the ECG wearables to ensure that they remained in place and functioned correctly (Figs. 1–3), adding to their cognitive load and overall discomfort. This increased attention could also be attributed to the sensory input produced by the ECG device, which heightened cognitive engagement^23^. These sensory stimuli from the wearables may also explain the participants’ reports that there was some impact on their sleep. Despite these challenges, the overall response to wearable technology was predominantly positive, suggesting a complex yet favorable reception in the community. These challenges indicate that there is room for refined wearable designs and technical improvement to minimize intrusion into daily life, reduce sleep disruption, enhance social and psychological comfort, and alleviate cognitive burden.

The variations we observed in acceptability based on sex and wearable type (Figs. 2 and 3) highlight the need for tailored deployment strategies that consider these individual differences. This finding is consistent with the literature that emphasizes the importance of addressing the unique needs of specific groups defined by sex, age, and other characteristics when deploying wearables^24,25^. Recognizing and accommodating individual preferences, even within a setting that prioritizes collective benefits, is key to the successful adoption and sustained use of wearable technologies in health research.

Our findings show high rates of data availability and completeness across the wearables (Fig. 4). Compared to consumer-grade wearables deployed in a similar context^16^, we noted greater data completeness for research-grade wearables, likely due to greater storage capacity. On the other hand, research-grade wearables are more expensive than other wearables and require more effort for data synchronization and post-processing. Our results align with those of Din et al. in 2020, who demonstrated low data loss (<9%) with a high-resolution wearable accelerometer for measuring walking activity in older, rural-dwelling adults in Tanzania^21^. Therefore, our findings demonstrate the feasibility of continuous individual and environmental monitoring in a real-world setting. This robust data collection capability positions research-grade wearables as promising tools for community-based health research in rural settings.

The data from the wearable devices showed that women experienced greater physiological and cardiovascular strain than men despite having similar physical activity and sleep patterns (Table 3 and Fig. 7). This finding suggests potential sex differences in experience and response to physical stress, which could inform sex-specific interventions. The model (Supplementary Fig. 1) indicated that an outdoor WBGT significantly increased the PSI (*β* = 0.32, *p* < 0.001), with males having a higher baseline PSI (*β* = 5.33, *p* < 0.001), but the effect of the WBGT on PSI was less in males than females (*β* = −0.27, *p* < 0.001). This analysis should be interpreted cautiously due to the study’s limited statistical power. Despite high physical activity (2.3–3.0 h of MVPA per day) and low sleep efficiency (61.8% for women, 59.7% for men), physical exertion may not translate into restful sleep, possibly due to factors such as heat stress impacting recovery and work capacity.

HR, ACT, and CBT data from the selected wearables allowed continuous monitoring over time (Fig. 7). These metrics, especially during physical effort, provide objective data on heat strain, enabling the use of indices such as the PSI^7^. PSI, originally developed for laboratory use, can be further modified for continuous monitoring in the field and to account for differences across populations^26,27^. In addition, by integrating ACT data (Fig. 8), the physiological and activity strain index (PASI) can account for both environmental heat and physical exertion^28^. GPS tracking (Supplementary Fig. 3) further enhances the understanding of subsistence farming work patterns, offering insights into the timing, duration, and location of physical activities, helping to assess labor capacity and develop adaptation strategies in the context of climate change.

Understanding the impact of sex on the adoption of wearable materials is critical, but it is also vital to consider sex-related differences in the response to heat stress. For example, as shown in Table 3, women exhibited a significantly greater physiological strain index (PSI) and greater relative change in heart rate (HR) than men did, which may indicate a heightened susceptibility to heat stress. This could be attributed to a combination of factors, including lower heart rate reserve capacity and increased exposure to physically demanding tasks, both in the home and on the farm, where women constitute more than 50% of the labor force. Notably, physical activity levels were high for both sexes (mean daily steps >10,000), and there was evidence of pacing, with most moderate-to-vigorous physical activity (MVPA) occurring in the morning and early afternoon before outdoor WBGT peaks (Figs. 5 and 6). Interestingly, women tend to reach their peak physical activity earlier than men do but sustain their effort until men’s peak, after which both groups experience a decline. These findings suggest that women may be at greater risk of heat-related strains due to both physiological differences and sustained manual labor; thus, there is a need for targeted interventions to mitigate heat stress, particularly for women in agricultural settings. One possible consideration is work pacing; a simple behavioral strategy that seems to be spontaneous in this setup where laborers are working on their own farms.

A limitation of this study is potential selection bias, as participants were from a specific geographic area and demographic group, limiting the generalizability of the findings within SSA. In addition, the small sample size and short follow-up limit inferences about the effects of heat stress on physiological parameters, activity, and sleep parameters. However, our approach of conducting a descriptive pilot study provides valuable insights for planning future analytical studies involving continuous environmental and physiological monitoring. Data collection challenges, such as device malfunctions due to power outages and compliance with continuous device use, could arise in longer studies, although we did not encounter these issues, likely due to the short study duration. Another key strength is the inclusion of women, who make up 50% of the agricultural workforce in SSA; this study addresses the significant gap in research on the impacts of heat stress on women workers^29^.

In conclusion, this study demonstrated that research-grade wearables are not only feasible but also highly acceptable in a rural Western Kenyan setting. However, given the study’s small sample size, appropriate for a pilot study but not for generalizable conclusions, these findings should be interpreted with caution. Further research is needed in larger and more diverse populations to confirm their broader applicability. These findings have significant implications for public health, particularly in the development of adaptive strategies to mitigate the health impacts of climate change. By providing real-time data on physiological and behavioral (physical activity and sleep patterns) responses to heat stress, these devices could play a crucial role in protecting vulnerable people from the adverse effects of rising temperatures. Future research should focus on expanding the use of wearable technology in other regions and populations within SSA, as well as developing targeted interventions that leverage the data generated by these devices to improve health outcomes.

## Methods

### Study setting and population

Within the KEMRI/CDC HDSS area, we conducted a pilot study from August to November 2021 with an observational time-series design. The KEMRI/CDC HDSS, located in Siaya County-western Kenya, is characterized by a hot and humid climate that is influenced by Lake Victoria. The area is predominantly rural, with residents relying on fishing and small-scale rain-fed agriculture, and a majority live in poverty^17^. Siaya County experiences two distinct rainy seasons, from March to May and October to December, significantly impacting agriculture and animal husbandry. We focused on this farming community due to their heightened vulnerability to hyperthermia and heat-related illnesses. This vulnerability stems from increased metabolic heat generation during strenuous physical activity and exposure to extreme heat, humidity, and solar radiation while working outdoors^3^. The study area covered a 5 km radius centered around the weather station in Wagai village, Siaya, which is part of the KEMRI/CDC HDSS. In addition, clinic-based procedures were carried out at the Wagai Health Center, a community health centre within the study area.

**Fig. 9:**
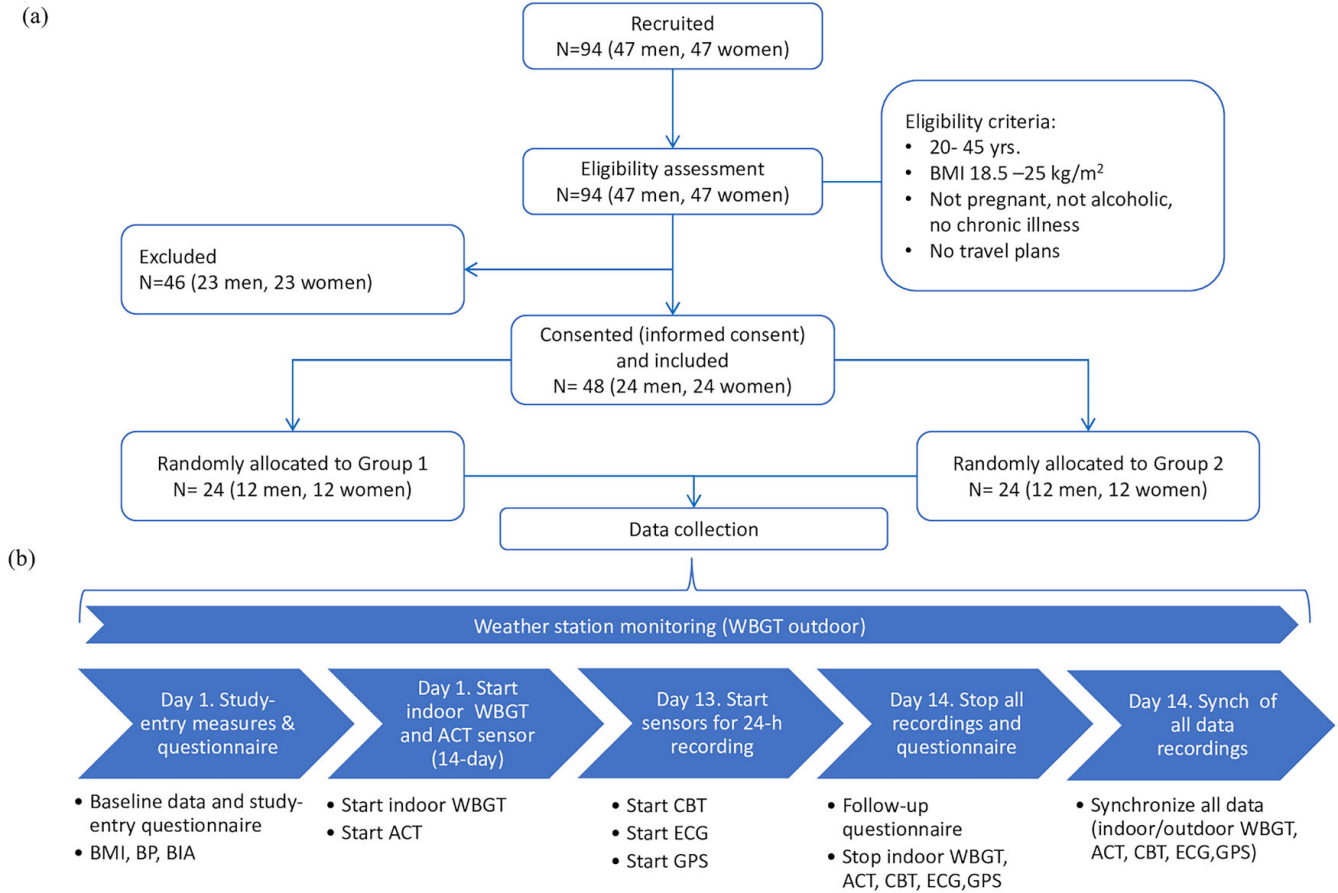
Overview of recruitment and study protocol. The flowchart in the upper panel (**a**) details the recruitment, screening, and group allocation activities within the study, including the number of participants at each step. The lower panel (**b**) provides a graphical description of the data collection procedures. BMI body mass index, BP blood pressure, BIA body impedance analysis, WBGT wet-bulb globe temperature index, ACT actigraphy, CBTcore body temperature, ECG electrocardiography, GPS location tracking with a Global Positioning System device.

We constructed a sampling frame using the HDSS database, which contains detailed information about individuals, such as age, sex, relationships, income sources, and current place of residence. From this database, we identified 156 couples who met our inclusion criteria. We then followed the study recruitment process and measurement procedures, as illustrated in Fig. 9. Individuals were eligible for the study if they met the following inclusion criteria: were (a) married or living together, (b) aged between 20 and 45 years, (c) relied on subsistence farming as their main source of income, and (d) resided within a 5 km radius of the automated weather station and the study clinic.

Focusing on farmers allowed us to monitor heat stress in open fields while targeting a working-age population with relatively consistent thermoregulatory responses. The minimum age of 20 excluded children and adolescents, who may not be able to provide independent consent and have different thermoregulatory responses—while the maximum age of 45 minimized confounding effects related to aging. In addition, we determined that the 5 km radius surrounding the weather station adequately covered local weather conditions. Male–female pairs were selected to explore sex-related differences in physiological responses to heat^30^. This selection also facilitated the analysis of how men and women may utilize devices differently, given that their daily routines can vary. Lastly, recruiting couples from the same household reduced cultural barriers, as women’s participation may depend on their partner’s approval.

We aimed for a sample size of 24 men and 24 women. Given the pilot nature of the study and the specificity of the inclusion criteria, the selected sample size was deemed sufficient. This approach allowed for an evaluation of the acceptability of the wearables and the data logger, their ease of deployment in a rural setting, and their ability to generate reliable, high-quality data under real-world conditions. Assessing data quality was essential due to known challenges with wearables, including data loss, adherence issues, and technical barriers^31,32^.

### Recruitment

Community health workers invited 156 eligible pairs to the study clinic, 94 of whom ultimately attended. We recruited male–female household pairs to ensure female participation and capture sex-related differences. In settings where cultural barriers limit women’s involvement, including men from the same household fosters an environment that supports and endorses female engagement, thereby enhancing our pilot study’s representativeness. After rescreening based on the eligibility criteria, we further excluded individuals with conditions impacting thermoregulation (e.g., pregnancy, cardiovascular conditions, metabolic diseases, and chronic obstructive pulmonary disease) or those affecting physical performance (e.g., body mass index (BMI) > 25 kg/m^2^), as well as individuals reporting alcohol abuse or imminent plans to out-migrate from the study area. Entire pairs were disqualified if either member was ineligible or withheld consent. All participants were informed about the study details and signed informed consent forms prior to participation (Ethical Approval Numbers: EA1/060/19 Charité - Universitätsmedizin and KEMRI/SERU/CGHR/327/3962-Kenya Medical Research Institute). To ensure participant privacy and confidentiality, all the collected data were pseudonymized, and stringent data security measures, including password protection, were implemented. Identifiable images were only included with written consent; in such cases, an opaque rectangular shape was placed over the face to ensure participant anonymity. After providing written consent, 24 male–female pairs (*n* = 48 participants) were enrolled and randomly divided into two groups, each undergoing data collection in separate phases. Participants in Group 2 began wearing their devices a week after Group 1, creating a 7-day gap between the groups. This approach was necessary for two reasons: (1) to allow the team to identify and address unexpected issues, as this was the first time research-grade wearables were used in this setting, and (2) the limited availability of wearable devices in this feasibility study made it impractical to monitor all participants simultaneously.

### Experimental protocol

**Fig. 10:**
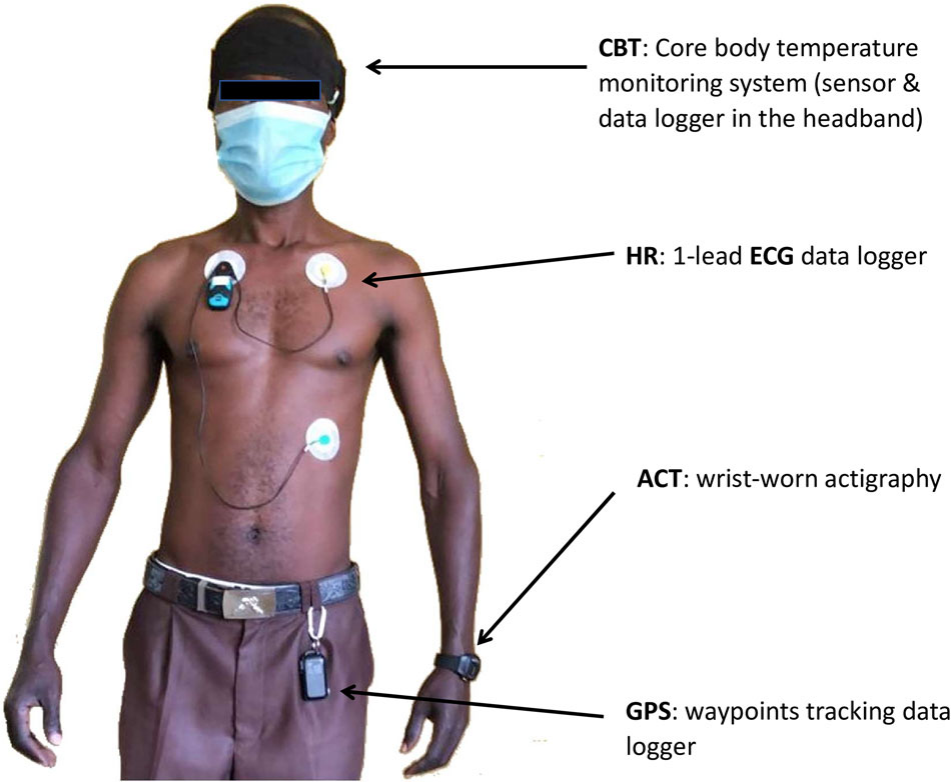
Placement of wearable devices. Example of a fully instrumented participant. The wearables are identified by the parameters they recorded: CBT (core body temperature), ECG (electrocardiography), ACT (actigraphy), and GPS (location tracking with a Global Positioning System device). Note: This figure includes an image of a participant taken with written consent; an opaque rectangular shape has been placed over the face to protect the individual’s identity.

At their first clinic visit, each pair underwent initial assessments, including height, weight, blood pressure, and bioimpedance analysis, by trained staff. Demographic and anthropometric data were recorded using a tablet-based computer-assisted personal interview (CAPI) instrument. Additionally, each participant received a wrist-worn actigraphy device to monitor physical activity (Table 4), and each pair received a portable datalogger to monitor indoor environmental conditions at home. The datalogger continuously monitored the indoor WBGT. On the thirteenth day, participants underwent 24-hour core body temperature monitoring via a head-worn wearable device and continuous electrocardiography with a chest-worn device to assess heart rate, and 22-hour individual location tracking was performed using a clothing-attached global positioning system device (Table 4 and Fig. 10). Participants were specifically encouraged to maintain their regular daily activities throughout the monitoring period. In addition, all wearable devices were configured using the manufacturer’s software and fully charged at the study clinic prior to deployment. Furthermore, to ensure accuracy, clocks across all devices were aligned with local time, allowing for precise synchronization of data from multiple sources. During data collection, the study team performed periodic checks to ensure device functionality and data integrity. Upon completion of the monitoring period, participants underwent a second round of the CAPI survey, which covered device acceptability, daily routines, device status, and procedural adherence. Participants were reimbursed for transportation costs, and all wearable and environmental monitoring data were securely downloaded onto a dedicated laptop for subsequent analysis.

**Table 4:** Device specifications

Device	(a) Tcore Sensor (b) Data logger and headband	Bittium Faros 180°	GENEActiv Original	Renkforce GP-102 G-Porter GPS Logger
Company	(a) Dräger, Lübeck, Germany (b) KORA, Hamburg, Germany	Bittium, Finland	Activinsights, UK	Renkforce, Germany
Image				
Measurement	CBT (core body temperature)	ECG (Electrocardiography)	ACT (Actigraphy)	GPS (Geospatial Position)
Type of sensor	Tcore sensor with heat-flux technology	1-lead mini-ECG with 3 electrodes and built-in tri-axial accelerometer	Tri-axial accelerometer, ambient light skin temperature sensors	GPS utilizing the MTK3339 chipset
Measurement features	Temperature Range: −30 °C to 45 °C	ECG Sampling Rate: up to 250 Hz; Accelerometer	Range: +/- 8 g	GPS Accuracy: Up to 2.5 meters
	Sampling Rate: 0.0167 Hz	Sampling Rate: 10 Hz	Sampling Rate: 10–100 Hz	Sampling Rate: 0.0033 - 1 Hz
Memory capacity	8 MB	Up to 64 GB	0.5 Gb	4MB flash memory
Data transfer	USB cable	Micro-USB cable	Cradle with USB cable	USB cable
Data format	LAB	EDF (European Data Format)	BIN (binary)	GPX, KML, CSV

Characteristics, functions, and features of selected wearables for physiological monitoring.

### Anthropometry and demographic baseline data

Participants’ heights were measured to a precision of 0.1 cm using a wall-mounted stadiometer and body weights were assessed with a calibrated scale accurate to 0.2 kg. From these, BMI was calculated. Bioimpedance analysis (BIA) for body composition was performed using a single-frequency BIA 101 analyzer (AKERN Srl, Florence, Italy) at 50 kHz. BIA was conducted according to the manufacturer’s guidelines^33^. Subsequently, total body water (TBW) and fat mass (FM) were computed employing an equation specifically developed and validated for adult populations of African descent ^34,35^. Furthermore, blood pressure was measured using a digital sphygmomanometer (Omron JPN 600) on the left arm of seated participants, who were allowed to rest upon arrival at the clinic. The duration of this rest period was determined based on participants’ feedback, as we did not establish a specific criterion for what constituted an ‘adequate rest’ period. All the baseline anthropometric data were collected in the study-entry questionnaire, described below.

### Questionnaires - Computer-assisted personal interviews

We administered two questionnaires (see Supplementary Material, Supplementary Study Questionnaires) deployed on the *Survey Solutions* Computer-Assisted Personal Interview (CAPI) platform^36^, i.e., a study-entry questionnaire and a follow-up questionnaire. The study-entry questionnaire captured the participants’ initial willingness to use the devices. In addition, to confirm adherence to the study protocols, we captured information on device management (charge, configuration, cleaning, and placement). For devices provided on day 13 of follow-up, device management data were collected on the material day.

The follow-up questionnaire was administered at the time of the study’s conclusion to gather participants’ perspectives on device acceptability. In this study, “acceptability” was defined as participants’ willingness to use the wearable devices, their perceived usefulness of the technology, and any physical, psychological, or social discomfort experienced while wearing the devices. This approach aligns with the Technology Acceptance Model (TAM), which highlights the perceived usefulness and ease of use as key factors influencing the adoption and continued use of new technology^20^. Participants responded to Likert scale items (ranging from “strongly agree” [1] to “strongly disagree” [5]) on topics such as device usefulness (“wearing made no sense”), psychological response (“happy to wear the device”, “strange to wear the device”), cognitive burden (“device requires too much attention”), physical burden (“cumbersome to wear”, “device limited my movements”), impact on daily routine (“wearing affected daily routine”, “wearing affected sleep”), and aesthetic appeal (“found the device likable”). Additional questions included binary (yes/no), multiple-choice, and open-ended formats. The staff also reported on tasks such as charging, configuration, cleaning, placement, data download, synchronization, and any instances of device loss or damage.

After completing the questionnaire, a trained operator verified the accuracy of the data, identifying and rectifying any discrepancies, such as duplicates or data entries. Subsequently, the verified questionnaire was integrated into the final dataset. The dataset was thereafter downloaded from the *Survey Solutions* server for further analysis. To assess the respondents’ feelings toward the wearables, we computed both the absolute and relative frequencies of the responses to each item in the questionnaire. Additionally, we conducted a sex-based stratified analysis to investigate potential differences. To visualize the Likert scale items, we utilized divergent bar graphs.

### Individual physiological monitoring

We measured 24-h CBT using the Tcore sensor (Dräger, Lübeck, Germany) sensor attached to a miniaturized data logger (KORA Homburg Germany)^37^. Both were embedded in a custom-made headband to improve wearability during daily activities (including farming). The 24-h HR was measured using a one-lead miniaturized electrocardiography (ECG) system (Faros 180°, Bittium, Oulu, Finland) with three electrodes attached to the thorax^38^. Furthermore, activity levels, including steps (ACTs), were continuously monitored using the GENEActiv Original watch (ActivInsights, UK) ^39^. The participants’ spatial movements were tracked for a 22-h span using a wearable GPS logger (GPS) (Renkforce GP-102, Conrad, Germany). Table 4 summarizes the key features of these four research-grade wearables, while Fig. 10 depicts their placement on the body. The ACT watch was placed on the non-dominant wrist and worn for 14 days. The GPS device was attached to a belt or cloth at the waistline and worn for 22 h.

To manage the devices efficiently, we utilized dedicated manufacturer-provided software on a laptop for wearable configuration, firmware updates, and secure data transfer. After downloading the data, a thorough first check for quality was performed before securely uploading the verified datasets to a protected server. After data synchronization through their respective native software packages on the study computer, the raw data from the wearable devices were postprocessed to derive meaningful physiological and activity metrics.

For ECG post-processing, we used Kubios Premium software (Kubios Oy, Kuopio Finland) for R-peak detection and heart rate extraction. Actigraphy data were processed using the GENEActiv R Markdown Analysis Tools, which are freely available online^40^; these tools segment raw acceleration, expressed in *g* units (gravitational acceleration)^41^; and ambient light signals into epochs classified as sedentary, light, moderate, or vigorous activity based on predefined acceleration thresholds from Esliger et al.^42^. This process yielded summary metrics such as daily step count, sleep duration, sleep efficiency, and time spent performing different activities.

We processed GPS waypoints to estimate the time spent in the field and working patterns by using farm-centroid coordinates to establish farm boundaries via the Haversine formula^43^. The average farm size of 1.2 acres^44^ was increased to 3 acres to account for GPS inaccuracies. GPS home positions were overlaid with waypoints to identify activity locations (home vs. farm vs. elsewhere), and we calculated the distance covered and the time spent in these areas.

The feasibility of research-grade wearables was evaluated based on the volume of good-quality, usable data each device produced. Usable data were obtained by excluding corrupted data and filtering out implausible values. Corrupted data refers to information that was unprocessable due to equipment malfunctions or transmission issues. Implausible data, such as heart rate below 40 bpm or above the maximum predicted by the Tanaka formula^45^ and CBT readings outside the range of 35 °C–42 °C, were treated as outliers^46,47^. Data availability was defined as the proportion of participants (*n* = 48) from whom at least one usable data recording was retrieved. On the other hand, the completeness of the data was calculated as the percentage of the total intended recording time, which was captured as usable data for each device worn by each individual. For example, if a device was set to measure heart rate continuously over a 24-h period, data completeness for a specific individual would reflect the proportion of that 24-h period during which usable heart rate data were successfully recorded.

### Environmental monitoring

The automatic weather station installed in the study area collected outdoor environmental data, including air temperature, rainfall, wind speed and direction, and solar radiation. Using these data, the WBGT (measured in °C) outdoors was estimated according to Carter et al.^48^. As described previously, the WBGT integrates all the environmental factors that could impact human health and physical/labor performance into a single index^48,49^. For indoor WBGT monitoring, we used a PCE-WB 20 SD device (PCE Deutschland GmbH, Germany). The data were logged every 10 min and stored on an integrated SD card. We securely placed the device at a height of 2 meters from the ground in the main room where the study participants slept. The device was positioned to ensure unobstructed sensor access and proper air circulation and to avoid direct sunlight or windows. At the end of the monitoring period (14 days), each SD card was read, and the raw data were synchronized on the study laptop for further processing. The availability of data availability for the indoor WBGT was defined as the proportion of households (*n* = 24) with at least one good-quality data recording. As for wearables, we defined the data completeness for each distinct WBGT device as the percentage of the total monitoring period (14 days) for which usable data were recorded.

### Statistics

The raw data were processed using R statistical software (version 4.3.1), where they were cleaned, filtered for outliers, and analyzed for relevant metrics. Anthropometric data were compared between men and women using unpaired *t*-tests (Student’s *t*-tests), while laboratory and outdoor WBGT data were compared using one-way ANOVA, with statistical significance set at *p* < 0.05. Acceptability ratings were reported as percentages and categorized by device type and sex. The data availability and completeness for each device are expressed as percentages. Furthermore, to display completeness percentages at the individual level and by device type, we used the median to represent central tendency and provided detailed variability using the minimum, maximum, and interquartile range (IQR).

The heat strain was calculated as follows: Cardiac strain was computed at every observation as the increase in heart rate from the resting heart rate relative to the heart rate reserve capacity (HRR). The resting heart rate (RHR) was defined as the minimum observed HR, while the HRR was the difference between the predicted peak HR (based on the Tanaka formula^45^) and the RHR. Thermal strain was calculated as the increase in core body temperature from baseline (minimum CBT) relative to reserve CBT capacity. Reserve CBT capacity was defined as the difference between the critical temperature of 39.5 °C and the baseline or nadir CBT. The physiological strain index (PSI) combines both cardiac and thermal strain, with each component weighted by 5 and added together to produce a score ranging from 0 to 10, where higher values indicate greater physiological strain^7^. Both cardiac strain (relative heart rate increase) and thermal strain (relative CBT increase) are expressed as percentages. Physical activity metrics included daily step counts and time spent at different levels of activity, including moderate-to-vigorous physical activity (MVPA). Sleep metrics included total time in bed, total time asleep, and sleep efficiency which was calculated as the percentage of total time asleep relative to total time in bed. These metrics are reported as the mean and (SD), if not otherwise stated. These means of these metrics were then compared between men and women using linear mixed-effects models adjusted for the WBGT. The equation for the linear mixed-effects model was as follows:

$${Y}_{{ij}}={\beta }_{0}+{\beta }_{1}\cdot {{Sex}}_{{ij}}+{b}_{i}+{\varepsilon }_{{ij}}$$

Here, *Y*_*ij*_ represents the metric (e.g., daily step counts) for individual *i* at time point *j*, with *β*_0_ as the intercept, *β*_1_ as the coefficient for the predictor variable Sex_*ij*_, *b*_*i*_ as the random effect for individual *i*, and ε_*ij*_ as the residual error.

We also fitted a linear mixed-effects model, analyzing the relationship between the PSI and outdoor WBGT, adjusting for sex, total MVPA in hours, previous night sleep in hours, BMI, and fat mass percentage, with individual variables serving as a random effect to account for variability. The data, restricted to observations from 6:30 AM to 6:30 PM, were modeled using maximum likelihood estimation. The model equation was as follows:

$$\begin{array}{c}PSI={\beta }_{0}+{\beta }_{1}\cdot {Sex}_{ij}+{\beta }_{2}\cdot {WBGT}+{\beta }_{3}\cdot {WBGT}\cdot {Sex}_{ij}\\ \qquad +{\beta }_{4}\cdot {MVPA}_{ij}+{\beta }_{5}\cdot {Previous}\,{Night}\,{Sleep}\,{Time}_{ij}\\ \qquad +{\beta }_{6}\cdot {BMI}_{ij}+{\beta }_{7}\cdot {Percent}\,{Fat}\,{Mass}_{ij}+{b}_{i}+{\varepsilon }_{ij}\end{array}$$

In the equation, *“ij”* refers to the individual (*i*) and observation (*j*) levels within the model, *b*_*i*_ is the random effect for individual *i*, and ε_*ij*_ is the residual error. The fixed effects estimates, 95% confidence intervals, and random effects are provided in the Supplementary Material, as this analysis was beyond the scope of this paper but allowed for the exploration of potential analyses for follow-up studies.

All data post-processing, analysis, and visualization were carried out using the R statistical software, version 4.3.1^50^. The linear mixed-effects models were fitted using the *lme4* R package in R.

## Supplementary information

Supplementary Material

## Data Availability

The datasets used and/or analyzed during the current study are available from the corresponding author upon reasonable request. Please reach out to the corresponding author DK (daniel-phillip-oluoch.kwaro@charite.de).

## Code availability

The underlying code for this study is not publicly available but may be made available to qualified researchers upon reasonable request from the corresponding author.
