# Supplementary material for: Acceptability and feasibility of research grade wearables for monitoring heat stress in Kenyan farmers
Source: NPJ Digit Med. 2025 May 7;8:255. doi: 10.1038/s41746-025-01601-6 (PMC12059195; doi:10.1038/s41746-025-01601-6)
Supplement: Supplementary file 1 — Supplementary Material [file 41746_2025_1601_MOESM1_ESM.pdf]

Supplementary Material

STUDY ENTRY QUESTIONNAIRE

This questionnaire is administered to participants at the outset of the study. It records demographic details such as age, gender, ethnicity, education level, and socioeconomic status. Additionally, it captures anthropometric data obtained during clinic visits, including measurements such as height, weight, body mass index , vital signs, and results of bio-impedance assessment.

A. Demographic Information

1. Unique Study Identifier: ___________________________
2. Sex: _____________________________
3. Date of Birth: ______________________
4. [Single selection] What is your highest level of attained education? _________________

   Options: 1: None, 2: Primary, 3: Secondary, 4: Higher Secondary, 5: University, 6: Refuse to answer

5. [Free text] In which village do you currently reside? _____________________
6. [Date: yyy/mm/dd] For your current residency period, when did you start living in this village? ___________________________

B. Anthropometric Information

Instruction: *Record health data including Blood Pressure, Heart Rate, anthropometry (height and weight), and BIA values. Ensure the participant is calm and rested before taking Blood Pressure measurements. Ask the subject to empty the bladder and lie down quietly for about 10 minutes before taking measurements.*

7. Weight (kilograms): _________________
8. Height (centimetres): __________________
9. Body Mass Index (BMI) [calculated field, BMI = weight / (height /100)^2]: ____________
10. Systolic Blood Pressure (mmHg): ________________
11. Diastolic Blood Pressure (mmHg): ________________
12. Heart Rate (beats per minute): __________________
13. Body Impedance Assessment (BIA):
    a. Please insert BIA Resistance value: _________________
    b. Please insert BIA Reactance value: __________________

FOLLOW-UP QUESTIONNAIRE

On day 14, the conclusion of the study follow-up, participants complete this questionnaire, which employs a variety of question formats, predominantly utilizing a 5-point Likert scale. This questionnaire records insights regarding participants' experiences with the devices, capturing factors influencing both initial and continued use of these sensors. Specifically, questions address perceived benefits and ease of use associated with each device. Perceived benefits encompass two main aspects: perceived usefulness, indicating whether the device is considered beneficial or sensible to wear, and enjoyment derived from wearing the device. Questions related to ease of use encompass various dimensions, including physical, psychological, cognitive, and social burdens associated with device usage. Additionally, participants provide insights into the device's integration into their daily routines.

A. Participant Identification:

1. Unique Study Identifier: ___________________________

B. Receiving Sensors

2. [Multiple selection] Which sensor are you receiving? ____________________

   Options: Faros ECG, 2. TCore, 3. GENEActiv watch, 4. GPS data logger, 5. WBGT indoor temperature

C. Interviewer Impression about the Participant's Reaction:

Instruction: *Please provide your first personal impression/evaluation of the participant in response to the following questions, specifically focusing on the participant's initial reaction to each sensor.*

- *Respond for each of the following device types: 1. Faros ECG, 2. TCore, 3. GENEActiv watch, 4. GPS data logger, 5. WBGT indoor temperature*
- *To answer the Likert scale questions, select from one of the following options: 1: Strongly agree, 2: Agree, 3: Undecided, 4: Disagree, 5: Strongly disagree*

3. He/she likes the device.

   Device type: ______________ Option: _____________ (5 points Likert scale)

   Comments (separately, for each device): _____________________

4. He/she wants to use the device to track his/her health status.

   Device type: ______________ Option: _____________ (5 points Likert scale)

   Comments (separately, for each device): _____________________

5. He/she easily accepts to wear/use the device.

Device type: ______________ Option: _____________ (5 points Likert scale)

Comments (separately, for each device): _____________________

6. He/she is happy with wearing the device.

Device type: ______________ Option: _____________ (5 points Likert scale)

Comments (separately, for each device): _____________________

7. He/she seems to be worried about wearing the device.

Device type: ______________ Option: _____________ (5 points Likert scale)

Comments (separately, for each device): _____________________

8. Please add any further general comment about the device.

   a. Device type: ______________________ Comment: _______________________

D. Participant's Experience of the Sensors:

Instruction: *Ask the questions in this section in regard to wearables/data logger returned by the participant. Ask the participant to share their experience and impressions regarding the use of each sensor.*

- *Respond for each of the following device types: 1. Faros ECG, 2. TCore, 3. GENEActiv watch, 4. GPS data logger, 5. WBGT indoor temperature*
- *To answer the Likert scale questions, select from one of the following options: 1: Strongly agree, 2: Agree, 3: Undecided, 4: Disagree, 5: Strongly disagree*

9. Your first impression of the device is positive.

Device type: ______________ Option: _____________ (5 points Likert scale)

10. Your first impression of the device is negative.

Device type: ______________ Option: _____________ (5 points Likert scale)

11. You were happy to wear the device.

Device type: ______________ Option: _____________ (5 points Likert scale)

12. You were disturbed by wearing the device.

Device type: ______________ Option: _____________ (5 points Likert scale)

13. You found the device easy to use/wear.

Device type: ______________ Option: _____________ (5 points Likert scale)

14. You found the device likeable.

Device type: ______________ Option: _____________ (5 points Likert scale)

15. You found the device interesting.

Device type: ______________ Option: _____________ (5 points Likert scale)

16. You found the device important for your health.

Device type: ______________ Option: _____________ (5 points Likert scale)

17. You found the device useful.

Device type: ______________ Option: _____________ (5 points Likert scale)

18. You found the device requiring too much attention and care.

Device type: ______________ Option: _____________ (5 points Likert scale)

19. You found the device annoying.

Device type: ______________ Option: _____________ (5 points Likert scale)

20. You found the device difficult to wear/use.

Device type: ______________ Option: _____________ (5 points Likert scale)

21. You found the device disturbing to wear/use.

Device type: ______________ Option: _____________ (5 points Likert scale)

22. You found the device cumbersome to wear/use.

Device type: ______________ Option: _____________ (5 points Likert scale)

23. You found the device strange to wear/use.

Device type: ______________ Option: _____________ (5 points Likert scale)

24. You found that wearing/using the device did not make sense.

Device type: ______________ Option: _____________ (5 points Likert scale)

25. [Multiple selection] (select any that apply) Indicate whether you experienced any of the following issues/problems or adverse reaction by wearing the device.

    Options: 1: Cutaneous rush, 2: Itch, 3: Sensor causing pain, 4:Limitation of movements, 5:Working disturbances, 6:Sleep disturbances, 7:Disturbances of your personal care daily routine, 8:Must frequently check and take care of the sensor

a. Faros ECG - ______________________________________
b. TCore - ___________________________
c. GENEActiv watch - ________________________________
d. GPS data logger - __________________________________
e. WBGT indoor temperature - ________________________

26. [Free text] (select one option) Please describe any other issues/problems or adverse reactions from wearing the device?
    a. Faros ECG - ______________________________________
    b. TCore - ___________________________
    c. GENEActiv watch - ________________________________
    d. GPS data logger - __________________________________
    e. WBGT indoor temperature - ________________________

27. Wearing the device affected your working/daily activity.

    Device type: _______________ Option: ______________ (5 points Likert scale)

28. Please add comments about working while wearing the device.
    a. Faros ECG - ______________________________________
    b. TCore - ___________________________
    c. GENEActiv watch - ________________________________
    d. GPS data logger - __________________________________
    e. WBGT indoor temperature - ________________________

29. During the day, while working, you forgot you were wearing the device.

    Device type: _______________ Option: ______________ (5 points Likert scale)

30. You had to interrupt your activities several times because of the device.

    Device type: _______________ Option: ______________ (5 points Likert scale)

31. You had to remove the device.

    Device type: _______________ Option: ______________ (5 points Likert scale)

32. The device was limiting your movements.

    Device type: _______________ Option: ______________ (5 points Likert scale)

33. You had to adjust/replace positioning of the device several times.

    Device type: _______________ Option: ______________ (5 points Likert scale)

34. The device was stuck on your skin because of sweating (in a disturbing way).

    Device type: _______________ Option: ______________ (5 points Likert scale)

35. You felt increased heat because of wearing the device.

Device type: ______________ Option: _____________ (5 points Likert scale)

36. [Single selection] (select one option) Did wearing the device have any effects on your sleep?
    Options: 1: Yes, 2: No, 3: I do not know

    Device type: ____________ Option: ____________

37. [Multiple selection] (select any that apply) How did wearing/using the device affect your sleep?

    Options: 1: sometimes I woke up, 2: I woke up frequently, 3: I could not sleep at all because of the sensor, 4: I felt tired in the morning, 5: The sensor fell off during the night, 6: I felt increased heat because of wearing the sensor

    Device type: ____________ Options: _______________

38. [Single selection] (select one option) Were you comfortable wearing the device in public?

    Options**:** 1: Yes, 2: No, 3: I do not know

    Device type: ____________ Option: ____________

39. [Single selection] (select one answer) Was wearing the device physically uncomfortable?

    Options**:** 1: Yes, 2: No, 3: I do not know

    Device type: ____________ Option: ____________

40. [Single selection] (select one option) I had to adjust the device frequently.

    Device type: ______________ Option: _____________ (5 points Likert scale)

41. [Single selection] (select one option) I felt increased heat on the head because of the headband.

    Device type: ______________ Option: _____________ (5 points Likert scale)

42. [Single selection] (select one option) The Tcore sensor got displaced frequently. _______

    Options: 1: Strongly agree, 2: Agree, 3: Undecided, 4: Disagree, 5: Strongly disagree

43. [Free text] Please describe any other negative experience with the Tcore device.
    ____

44. [Single selection] (select one option) Was wearing the Faros ECG device physically uncomfortable? _______________________

    Options: 1: Yes, 2: No, 3: I do not know

45. [Single selection] (select one option) I had to fix or reposition the ECG electrodes frequently. ____________

    Options: 1: Strongly agree, 2: Agree, 3: Undecided, 4: Disagree, 5: Strongly disagree

46. [Single selection] (select one option) The ECG fell out frequently. _______________

    Options: 1: Strongly agree, 2: Agree, 3: Undecided, 4: Disagree, 5: Strongly disagree

47. [Single selection] (select one option) I used new ECG electrodes because they were not sticking or fell off.

    Options: 1: Strongly agree, 2: Agree, 3: Undecided, 4: Disagree, 5: Strongly disagree

48. [Single selection] (select one option) The ECG cables disturbed me or limited my working activities.

    Options: 1: Strongly agree, 2: Agree, 3: Undecided, 4: Disagree, 5: Strongly disagree

49. [Free text] Please describe any other negative experience with the ECG device. ____

50. Did people ask you about the device?

    Options**:** 1: Yes, 2: No, 3: I do not know

    Device type: ____________ Option: ____________

51. Did the device become a topic of conversation?

    Options**:** 1: Yes, 2: No, 3: I do not know

    Device type: ____________ Option: ____________

52. [Free text] Please describe what other people said to you about each device? ______________

53. [Free text] Please add any further comments regarding each device. ____________

54. In summary, did wearing devices cause a change in your usual daily habits?

    Options**:** 1: Yes, 2: No, 3: I do not know

    Device type: ____________ Option: ____________

55. [Multiple selection] (Select any that apply) How did wearing the sensors change your daily activities?

    Options: 1: Limiting my social activities, 2: Limiting my working activities, 3: Affecting my sleep (disturbing),4: Scarce social acceptance, 5: Adverse physical reactions (rush etc), 6: Others (describe)

    Device type: ____________ Options: ____________

56. [Free text] What would need to change for you to develop the habit of using these wearable devices or engaging with them? _________________

57. [Free text] Please describe any additional overall comment about negative experiences with the devices in general. _______________________

58. [Free text] Please add any comments, whether negative, positive, or neutral, with respect to wearable sensors. __________________________________

59. [Multiple selection] If you had to wear this sensor for a longer time period, for example months, what would be the barriers to participating in this study for you?

    Options: 1: Length of time required to wear, 2: Interaction level required (i.e., taking care of the sensor), 3: Desired sensor feedbacks (i.e., getting information from the sensor) , 4: Adverse effects, 5: Social acceptance, 6: Disturbance of daily activity, 7: Disturbance of sleep, 8: Disturbance of personal hygiene routine, 9: Other

    Device: ___________________ Options: ___________________

60. Did you experience any issues having the WBGT device at your home?

    Options**:** 1: Yes, 2: No, 3: I do not know

    Device type: ____________ Option: ____________

61. Did you experience any malfunctioning with WBGT at your home?

    Options**:** 1: Yes, 2: No, 3: I do not know

    Device type: ____________ Option: ____________

SUPPLEMENTARY RESULTS

Supplementary Figure 1: 24 hours WBGT and physiological variables from a Single Participant. Simultaneous data across a 24-hour period for a single study participant (woman): Panel (a) shows line graphs of indoor (portable data logger at home) and outdoor (weather station) Wet Bulb Globe Temperature (WBGT) in degrees Celsius (°C). Panels (b) and (c) present line graphs for Core Body Temperature (CBT) in degrees Celsius (°C) and Heart Rate (HR) in beats per minute (bpm), respectively, where data points are aggregated every 5-minute epoch to compute mean values. The gray band in the background of each graph represents the standard deviation of these 5-minute epochs.

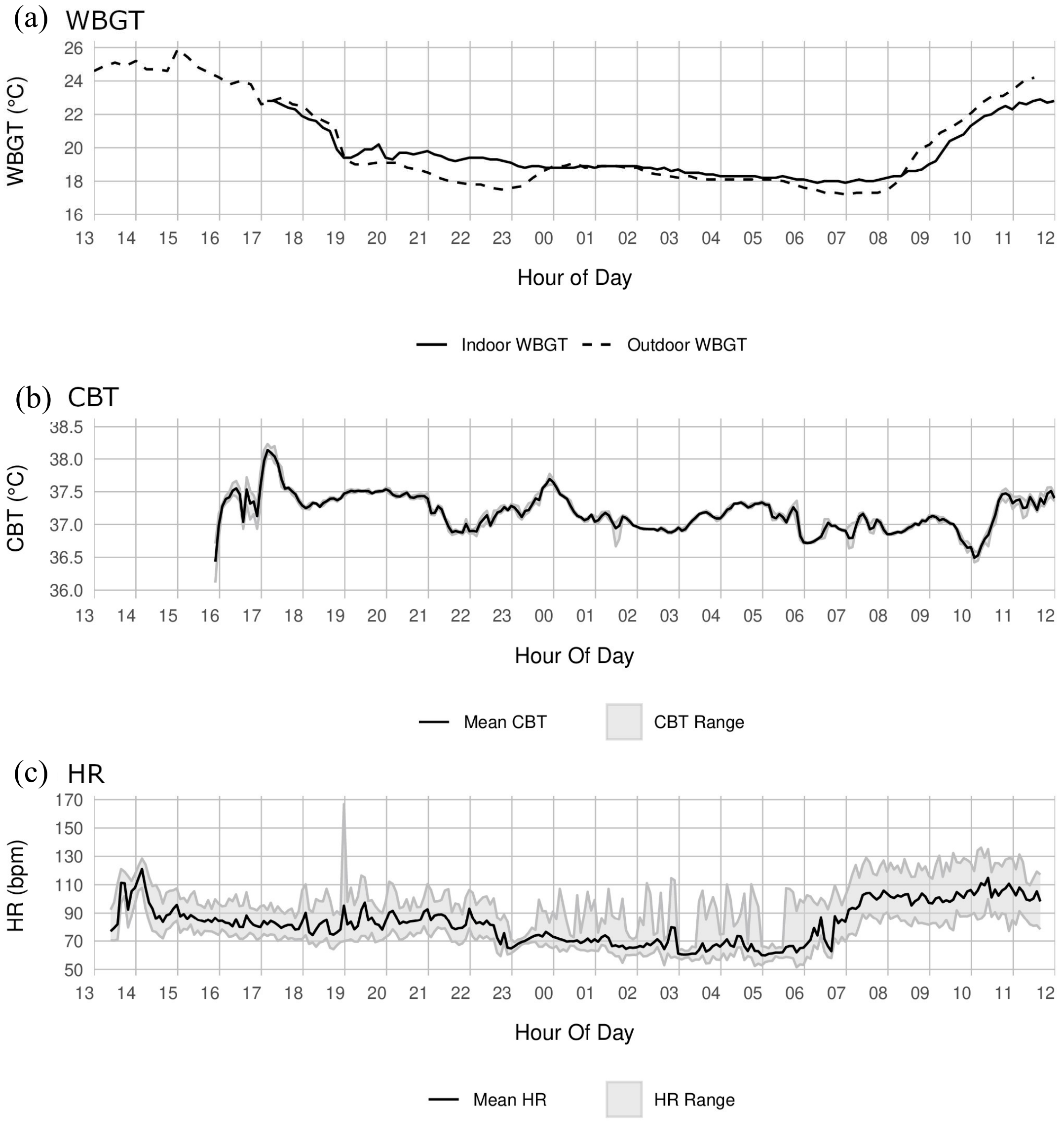

Supplementary Figure 2: Illustration of 14-day Synchronized Actigraphy Data Outputs for a Single Participant. Panels (a) through (d) showcase various parameters derived from the 14-day continuous actigraphy measurements. Panel (a) depicts a stacked bar graph illustrating the trend in the proportion of time spent on different activity intensity levels. Panel (b) presents a lollipop plot illustrating the variation in daily step counts. Panel (c) shows a line plot demonstrating the trend in total sleep time during the night. Panel (d) displays a line plot showcasing the trend in nightly sleep efficiency.

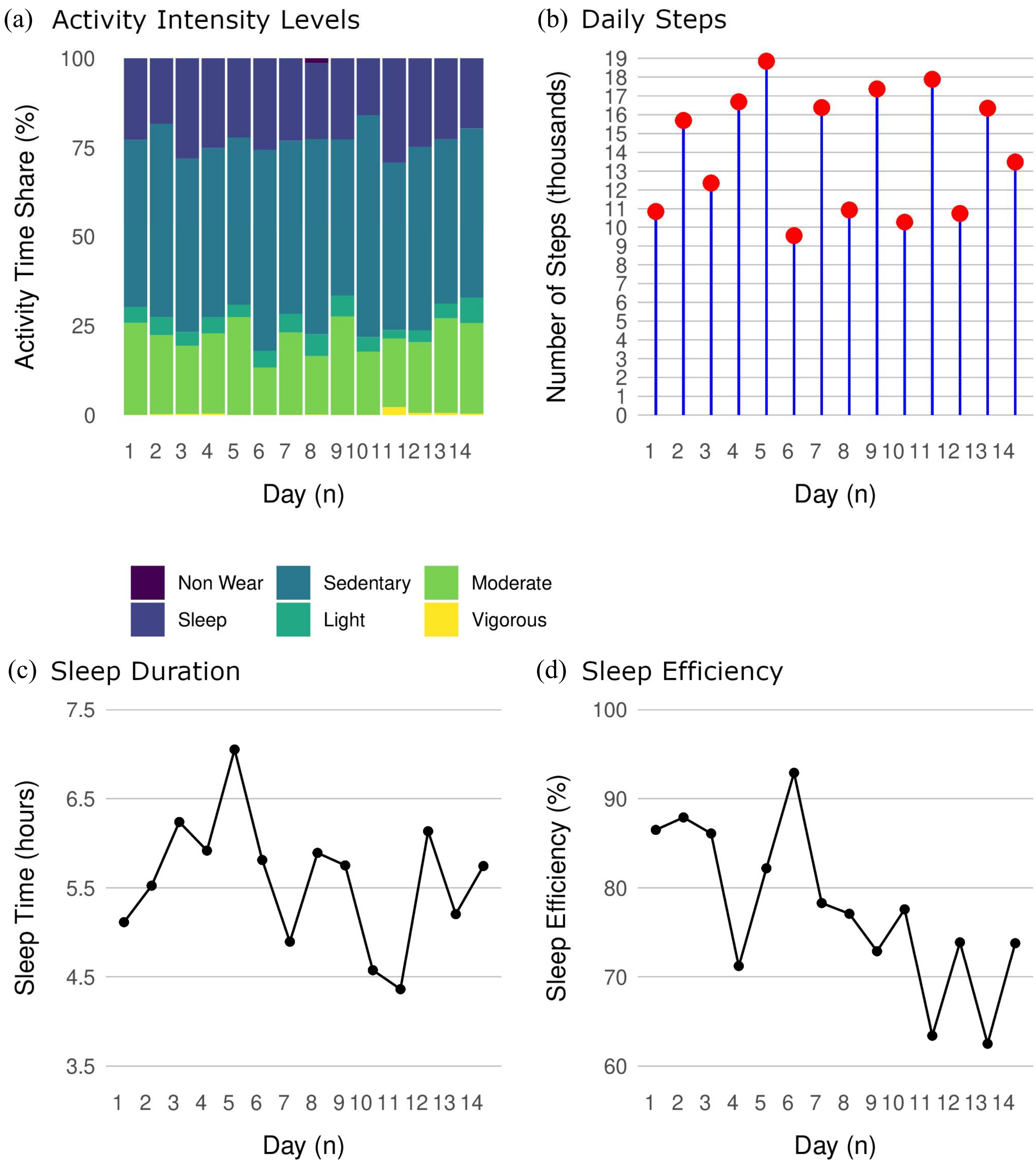

Supplementary Figure 3: Illustration of Movement Trajectory for a Single Case. Way points are connected to form tracks. This individual belongs to a household owning two farms. Acronyms: hrs- hours, km-kilometers.

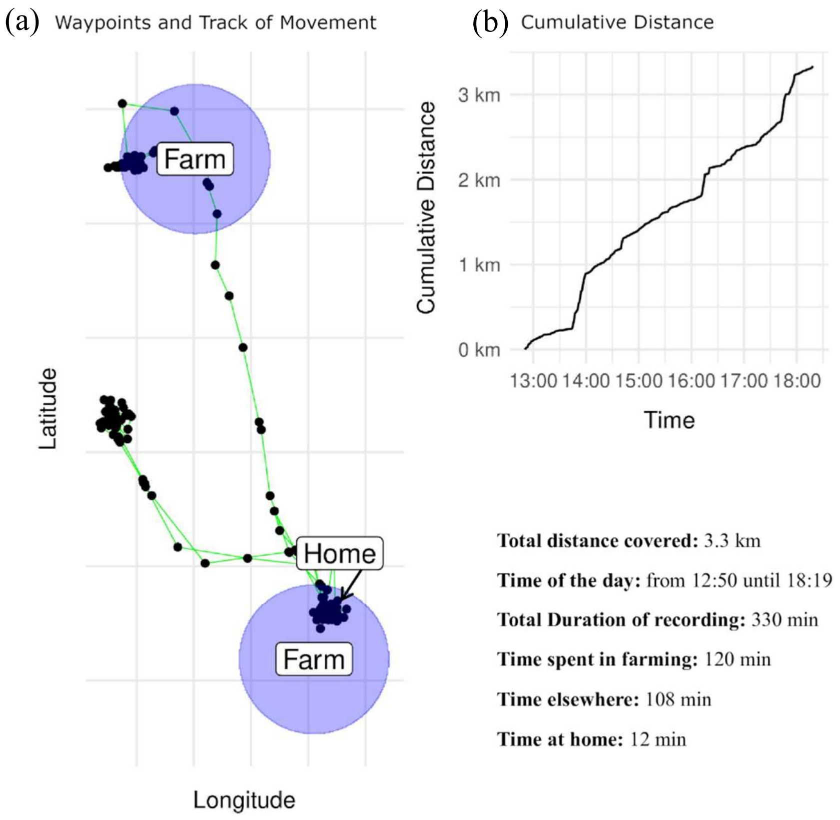

Supplementary Figure 4: Density Distributions of Physical Activity and Sleep Metrics Stratified by Sex. This multi-panel plot presents the density distributions of four metrics for males (red) and females (blue). Panel (a) shows the distribution of daily steps, while panel (b) displays Moderate-to-Vigorous Physical Activity (MVPA) hours. Panel (c) illustrates the distribution of total sleep time (hours), and panel (d) represents sleep efficiency (%).

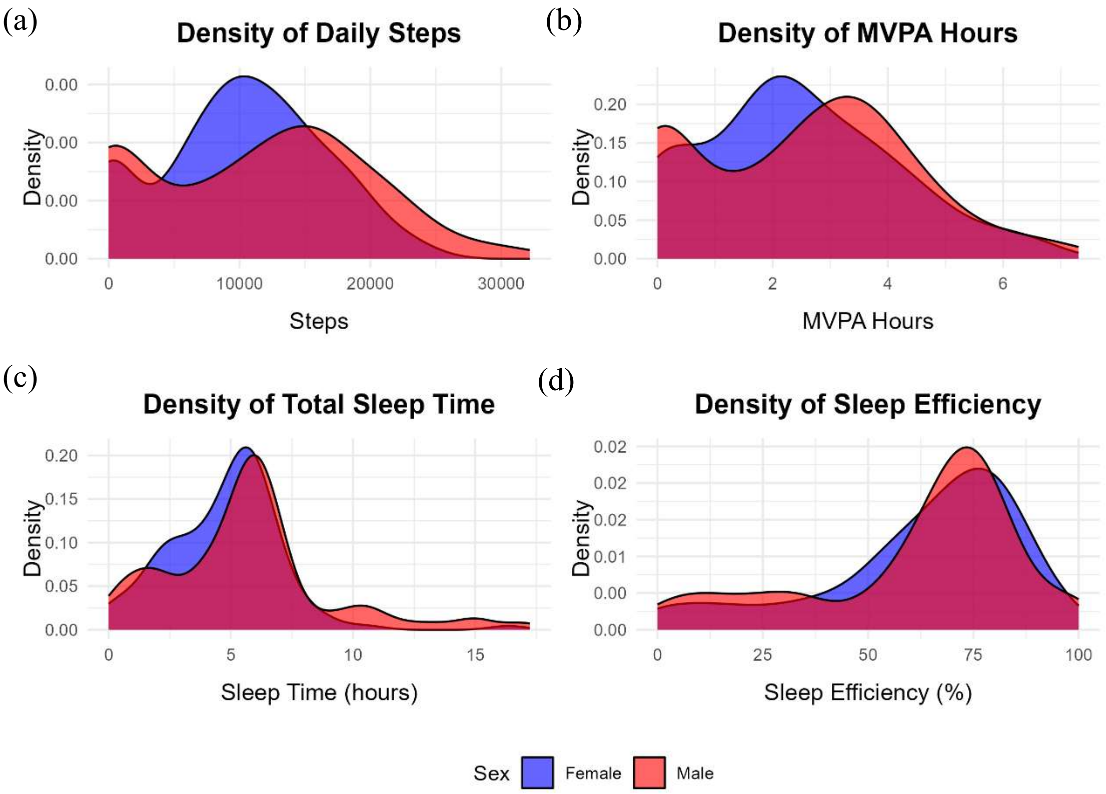

Supplementary Figure 5: Density Distributions of Heart Rate Metrics Stratified by Sex. This multi-panel plot presents the density distributions of six heart rate (HR) metrics for males (red) and females (blue). Panel (a) shows the distribution of Mean Heart Rate (beats per minute, bpm), while panel (b) displays Resting Heart Rate (bpm), highlighting baseline heart rate differences. Panel (c) focuses on Peak Heart Rate (bpm), and panel (d) illustrates Heart Rate Reserve (HRR), calculated as the difference between predicted maximum HR (using the Tanaka formula) and resting heart rates. Panel (e) represents the distribution of Relative Change in HR (%), showing HR fluctuations relative to HRR, and panel (f) presents the Coefficient of Variation in HR (%), which highlights heart rate variability. Heart rate (HR) was capped at a minimum of 40 bpm, and the predicted maximum HR was calculated using the Tanaka formula: 208−(0.7×age).

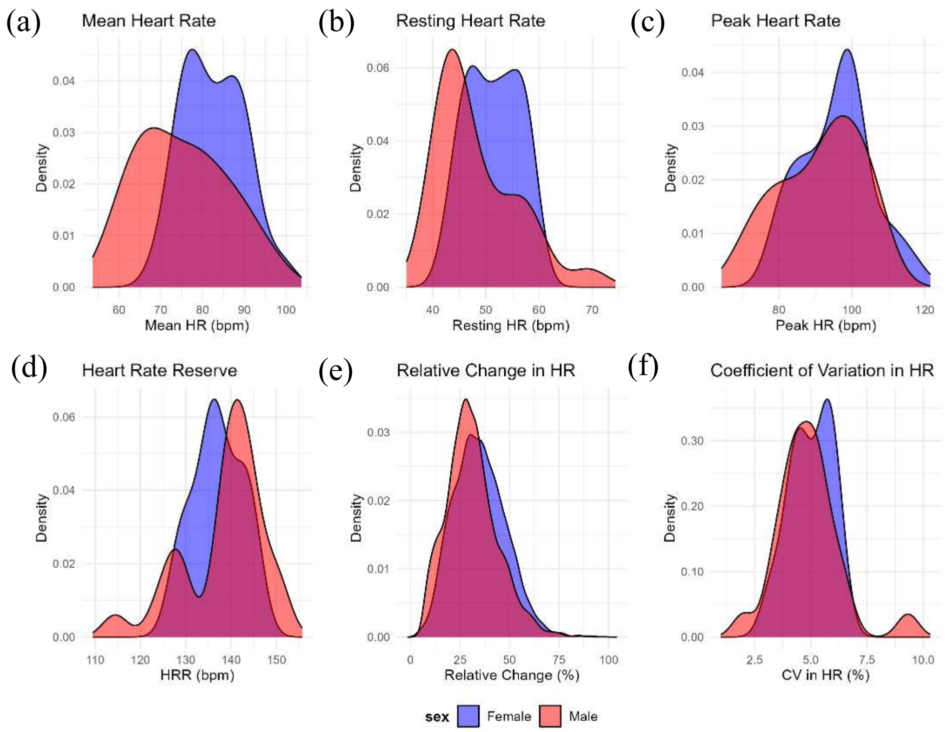

Supplementary Figure 6: Density Distributions of Core Body Temperature Metrics Stratified by Sex. This multi-panel figure shows density plots of core body temperature (CBT) metrics by sex (female in blue and male in red) for several physiological parameters. (a) Mean CBT: The overall average CBT distribution, (b) Resting CBT: The minimum CBT during resting states, (c) Peak CBT: The maximum CBT recorded, (d) CBT Reserve Capacity: The difference between a maximum reference temperature (39.5°C) and the resting CBT, (e) Relative Change in CBT: The percentage change in CBT relative to the reserve capacity, and (f) Coefficient of Variation (CV) in CBT: The variability in CBT relative to the mean.

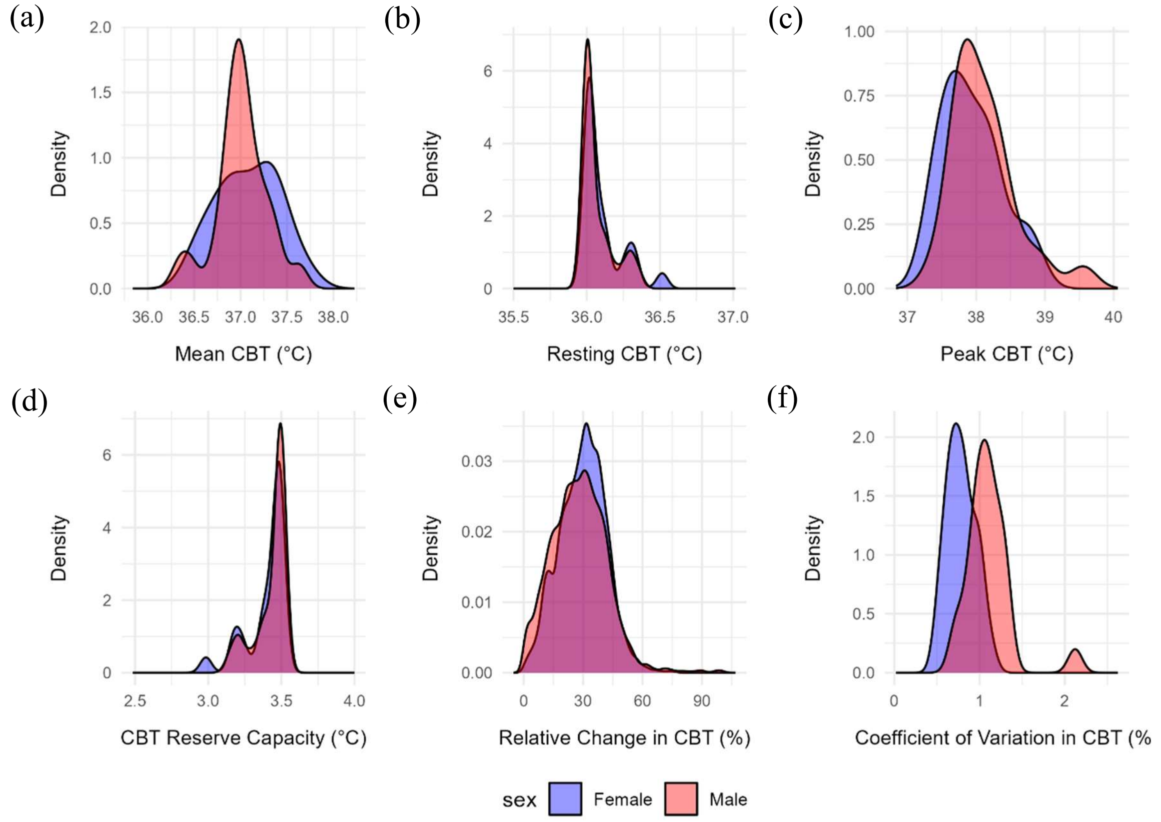

Supplementary Figure 7: Comparison of Physiological Strain Index (PSI) between Females and Males. Panel (a) shows the density distribution of PSI for females (red) and males (blue), indicating the range of strain levels experienced by each group. Panel (b) displays the percentage of individuals who experienced each PSI level (rounded to the nearest integer) for females and males.

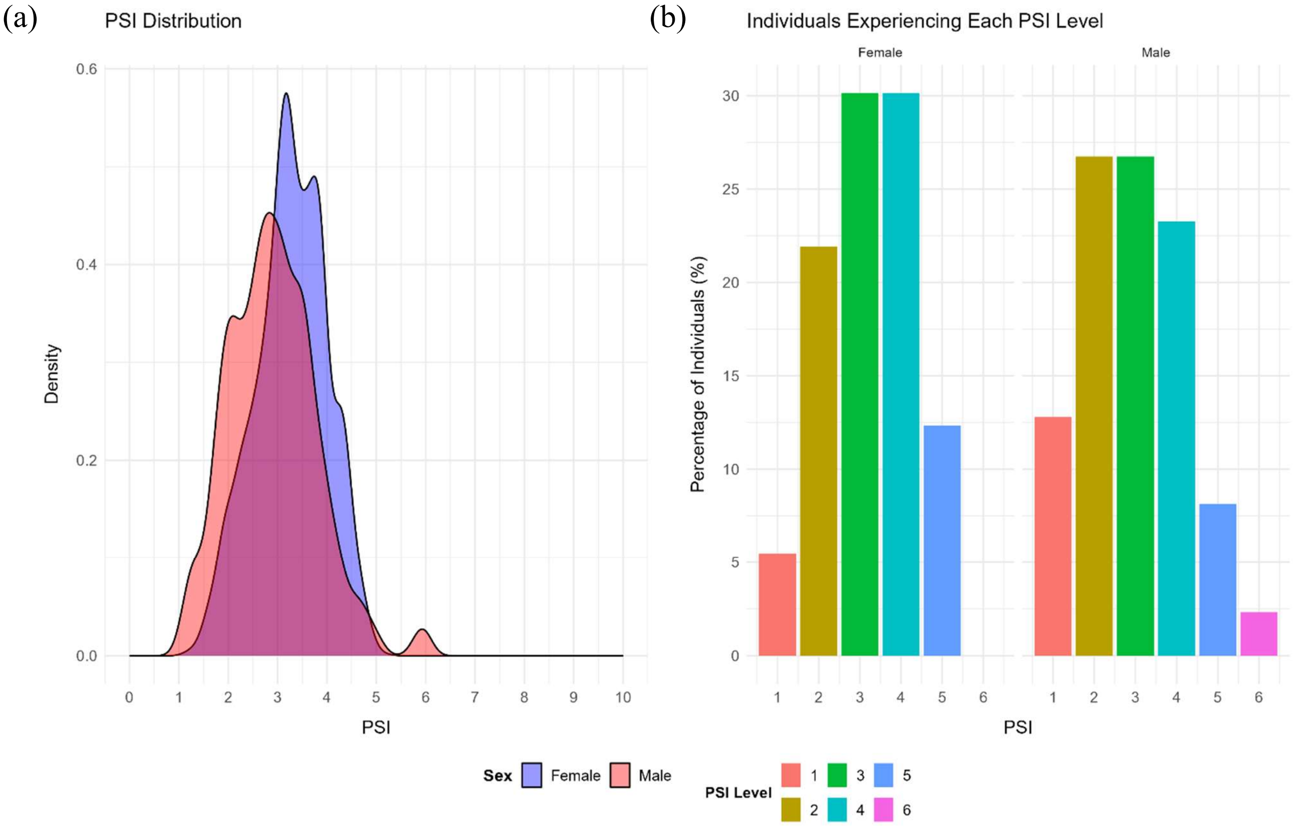

Supplementary Table 1: Effect of Outdoor WBGT on Physiologic Strain Index (PSI)
This table presents the fixed and random effect estimates from the linear mixed-effects model predicting Physiologic Strain Index (PSI). The model includes predictors for Total MVPA Hours, Previous Night Sleep Hours, Outdoor WBGT, BMI, Fat Mass Percentage, and Sex (Male), with individual as a random effect. Fixed effect estimates are provided with their corresponding standard errors, p-values, and significance levels. Random effects are represented by their standard deviations for the intercept and individual-level variability. Significance codes: *** $p < 0.001$, ** $p < 0.01$, * $p < 0.05$.

**Dependent Variable: Physiologic Strain Index (PSI)**			
**Predictor**	**Estimate**	**Std. Error**	**p-value**
Intercept	-4.393	1.464	0.009
Outdoor WBGT	0.320	0.029	<0.001 ***
Sex (Male)	5.328	0.891	<0.001 ***
Total MVPA (Hours)	-0.158	0.049	0.002 **
Previous Night Sleep (Hours)	0.107	0.094	0.271
BMI	0.045	0.108	0.682
Fat Mass Percentage	-0.009	0.071	0.905
Outdoor WBGT × Sex (Male)	-0.274	0.034	<0.001 ***
Random Effects:			
Individual ID (Intercept): Variance = 0.283, Std. Dev. = 0.533			
Residual Variance: Variance = 0.320, Std. Dev. = 0.565			
Conditional R^2: 0.68			
Marginal R^2: 0.40			
Notes: *Data filtered for observations between 6:30 AM and 6:30 PM.*			
* $p < 0.05$, ** $p < 0.01$, *** $p < 0.001$			

Supplementary Table 2. Linear Mixed-Effects Model for the Effects of Maximum Daytime WBGT on Daily Steps (in 1000s). This table summarizes the results from a linear mixed-effects (LME) regression model analyzing the impact of Maximum Daytime Wet Bulb Globe Temperature (WBGT, °C) on daily steps (in thousands). The model includes random effects for individuals to account for within-subject variability. Fixed effects predictors include Maximum Daytime WBGT (centered), sex (male), previous night's sleep duration (centered, in hours), and BMI (centered, in kg/m²). Centering refers to subtracting the mean of a predictor from each individual value, helping to interpret the coefficients in relation to the average value of the predictors. An interaction term between Maximum WBGT and sex (male) is also included. The standard deviations of the random effects (intercept and residual) are reported. Significant predictors are indicated with significance codes: *** p < 0.001, ** p < 0.01, * p < 0.05, and p < 0.1.

**Dependent Variable: Daily Steps (in Thousands)**				
**Predictor**	**Estimate**	**Std. Error**	**P-Value**	
Intercept	12.0	1.52	0.000	***
Max WBGT Daytime (°C, Centered)	-0.1	0.5	0.877	
Sex (Male)	1.5	2.2		
Previous Night Sleep (Hours, Centered)	-0.0	0.1	0.994	
BMI (kg/m², Centered)	-0.7	0.4	0.136	
Max WBGT Daytime (°C) * Sex (Male)	-0.5	0.8	0.577	
sd (Intercept)	4.7			
sd Observation	2.6			

Significance codes: '***' < 0.001, '.' < 0.1

Supplementary Table 3: Effect of Maximum WBGT During the Day on Moderate-to-Vigorous-Physical-Activity (MVPA)

This table presents the fixed and random effect estimates from the linear mixed-effects model predicting total MVPA hours. The model includes predictors for Previous Day MVPA (centered), Previous Night Sleep (centered), Max WBGT Daytime (centered), BMI (centered), and sex (Male), with individual ID as a random effect. Fixed effect estimates are provided with their corresponding standard errors, p-values, and significance levels. Random effects are represented by their standard deviations for the intercept and observation-level variability. Significance codes: *** p < 0.001, . p < 0.1.

Dependent Variable: Moderate-to-Vigorous-Physical-Activity (MVPA) in Hours

Predictor	Estimate	Std..Error	P.Value	
Intercept	3.23	0.47	0.000	***
Previous Day MVPA (Centered)	-0.17	0.09	0.057	.
Previous Night Sleep (Centered)	0.05	0.06	0.400	
Max WBGT Daytime (Centered)	0.02	0.14	0.899	
BMI (Centered)	-0.20	0.15	0.192	
Sex (Male)	-0.23	0.73	0.754	
sd (Intercept)	4.70			
sd Observation	2.60			

Significance codes: '***' < 0.001, '.' < 0.1

Supplementary Table 4: Linear Mixed-Effects Model for the Effects of Maximum Daytime WBGT on Total Sleep Time

This table presents the fixed and random effect estimates from the linear mixed-effects model predicting total sleep time in hours. The model includes predictors for Maximum WBGT Nighttime (centered), sex (Male), Total MVPA hours (centered), BMI (centered), and age (centered), with individual ID as a random effect. Fixed effect estimates are provided alongside their standard errors, p-values, and significance levels. The standard deviations of the random effects (intercept and observation-level variability) are also reported. Significance codes: *** p < 0.001, ** p < 0.01, * p < 0.05, . p < 0.1.

Dependent Variable: Total Sleep Time

Predictor	Estimate	Std..Error	P.Value	
Intercept	4.83	0.65	< 0.001	***
Max WBGT Nighttime (Centered)	0.05	0.18	0.781	
Sex (Male)	0.03	0.98	0.979	
Total MVPA Hours (Centered)	0.13	0.12	0.277	
BMI (Centered)	-0.03	0.18	0.865	
Age (Corrected, Centered)	0.06	0.09	0.506	
Max WBGT Nighttime (Centered) × Sex (Male)	-0.30	0.26	0.243	
sd__(Intercept)	0.90			
sd__Observation	0.35			

Significance codes: '***' < 0.001, '**' < 0.01, '*' < 0.05, '.' < 0.1
